# Thresholds for surfactant use in preterm neonates: a network meta-analysis

**DOI:** 10.1136/archdischild-2022-324184

**Published:** 2022-12-09

**Authors:** Aoife Branagan, Ivan Yu, Kurinchi Gurusamy, Jan Miletin

**Affiliations:** 1 Paediatric and Newborn Medicine, Coombe Women and Infants University Hospital, Dublin, Ireland; 2 Division of Surgery and Interventional Science, UCL, London, UK; 3 Department of Therapy, I.M. Sechenov First Moscow State Medical University, Moskva, Russian Federation; 4 UCD School of Medicine, University College Dublin, Dublin, Ireland; 5 Institute for the Care of Mother and Child, Prague, Czech Republic; 6 2nd Faculty of Medicine, Motol University Hospital, Prague, Czech Republic

**Keywords:** Intensive Care Units, Neonatal, Neonatology, Respiratory Medicine

## Abstract

**Objective:**

To perform a network meta-analysis of randomised controlled trials of different surfactant treatment strategies for respiratory distress syndrome (RDS) to assess if a certain fraction of inspired oxygen (FiO_2_) is optimal for selective surfactant therapy.

**Design:**

Systematic review and network meta-analysis using Bayesian analysis of randomised trials of prophylactic versus selective surfactant for RDS.

**Setting:**

Cochrane Central Register of Controlled Trials, MEDLINE, Embase and Science Citation Index Expanded.

**Patients:**

Randomised trials including infants under 32 weeks of gestational age.

**Interventions:**

Intratracheal surfactant, irrespective of type or dose.

**Main outcome measures:**

Our primary outcome was neonatal mortality, compared between groups treated with selective surfactant therapy at different thresholds of FiO_2_. Secondary outcomes included respiratory morbidity and major complications of prematurity.

**Results:**

Of 4643 identified references, 14 studies involving 5298 participants were included. We found no statistically significant differences between 30%, 40% and 50% FiO_2_ thresholds. A sensitivity analysis of infants treated in the era of high antenatal steroid use and nasal continuous positive airway pressure as initial mode of respiratory support showed no difference in mortality, RDS or intraventricular haemorrhage alone but suggested an increase in the combined outcome of major morbidities in the 60% threshold.

**Conclusion:**

Our results do not show a clear benefit of surfactant treatment at any threshold of FiO_2_. The 60% threshold was suggestive of increased morbidity. There was no advantage seen with prophylactic treatment. Randomised trials of different thresholds for surfactant delivery are urgently needed to guide clinicians and provide robust evidence.

**PROSPERO registration number:**

CRD42020166620.

**WHAT IS ALREADY KNOW ON THIS TOPIC**

- Intratracheal surfactant, provided to premature infants with neonatal respiratory distress syndrome (RDS), decreases mortality and the respiratory complications of prematurity.
- Current best practice supports nasal continuous positive airway pressure (NCPAP) and avoidance of mechanical ventilation, with provision of exogenous surfactant with increasing oxygen requirement or need for ventilation.
- Due to insufficient available evidence, clinical guidelines and therefore practice on when surfactant should be provided to these infants vary.

**WHAT THIS STUDY ADDS**

- This study adds to a limited evidence base on when is most appropriate to provide selective surfactant to infants with RDS.
- A threshold of 60% fraction of inspired oxygen has been shown to increase major morbidity, most notably retinopathy of prematurity, and should be avoided.
- No significant difference was seen between the 30%, 40% and 50% thresholds, which suggests more judicious use of surfactant may be appropriate.

**HOW THIS STUDY MIGHT AFFECT RESEARCH, PRACTICE OR POLICY**

- The results of this study suggest that more judicious use of selective surfactant may be appropriate in premature infants managed on NCPAP.
- Well designed and adequately powered randomised trials are required to further evaluate the most appropriate threshold of oxygen to provide surfactant to these infants.

## Introduction

Respiratory distress syndrome (RDS) is a common consequence of prematurity.^1^ Management is through provision of respiratory support alongside exogenous surfactant.^2^

Early Cochrane reviews supported prophylactic surfactant and intubation.^3^ A more recent review compared a prophylactic strategy (administration before first breath or after brief stabilisation) to selective use (after evidence of RDS), including subgroup analysis of current best practice (nasal continuous positive airway pressure (NCPAP) and high antenatal steroid use).^4^ The risk of chronic lung disease (CLD)/death was lower in the selective group in the subgroup supporting more judicious use.

Best practice dictates stabilisation of preterm infants with NCPAP and early surfactant if the need for intubation arises. However, the threshold at which this should occur is unclear. Despite a large body of work assessing the best use of surfactant, little work has assessed the threshold of fraction of inspired oxygen (FiO_2_) that surfactant should be given at, leading to variations in practice and reliance on poor quality evidence.^5 6^

Differing views exist internationally. The European Consensus Guidelines suggest a 30% threshold.^2^ Both the American Academy of Paediatrics and the National Institute for Health and Care Excellence (UK) state surfactants should be selectively given to infants on NCPAP but do not include a FiO_2_ threshold.^7 8^ More recently, the Canadian Paediatric Society suggested 50%.^9^ The value of FiO_2_ in isolation as a measure of RDS severity and surfactant requirement has been disputed, as FiO_2_ is influenced by multiple factors and pathologies.

Our aim was to perform a systematic review and network meta-analysis comparing different thresholds of FiO_2_ for surfactant treatment in infants under 32 weeks of gestation.

## Methods

A systematic review and network meta-analysis was conducted following Preferred Reporting Items for Systematic Reviews and Meta-Analyses (PRISMA) standards and was registered with the International Prospective Register of Systematic Reviews (PROSPERO) before commencement (CRD42020166620).

Network meta-analysis allows indirect comparison of data across studies. In the absence of direct evidence comparing thresholds of FiO_2_, it allows indirect comparison of intervention arms of trials which compare prophylaxis (control) and selective (intervention) treatment. As selective surfactant was provided at different thresholds of FiO_2_ in these trials, we can compare thresholds.

### Criteria for considering studies

#### Studies

Randomised controlled trials (RCTs) were considered, irrespective of language, publication status or publication date.

#### Participants

The participants included neonates from RCTs born before 32 weeks of postmenstrual age.

#### Interventions

Intratracheal surfactant delivery.

#### Outcomes

The primary outcome was mortality.

Secondary outcomes included

- Bronchopulmonary dysplasia (BPD) (oxygen requirement or need for respiratory support at 36 weeks of corrected gestational age (CGA))^10^
- CLD (oxygen requirement or need for respiratory support at 28 days).^10^
- Pneumothorax (or other air leak).
- Surfactant therapy (proportion requiring surfactant and number of doses required)
- Major morbidity, defined as at least one of severe intraventricular haemorrhage (IVH) (grade 3 or 4),^11^ periventricular leucomalacia (PVL),^12^ necrotising enterocolitis (NEC) (stage 2A or above),^13^ retinopathy of prematurity (ROP) greater than stage 2^14^ or BPD.
- Neurodevelopmental outcome at 2 years of CGA, defined as one of cerebral palsy, mental retardation (Bayley Scales of Infant Development Mental Developmental Index <70), legal blindness (<20/200 visual acuity) and hearing deficit (aided or <60 dB on audiometric testing).
- Health-related quality of life (HRQOL).^15^

### Search methods

Regarding electronic searches, we searched Cochrane Central Register of Controlled Trials (CENTRAL), MEDLINE (PubMed), Embase and Science Citation Index Expanded between inception and December 2021 without language restrictions.

We also searched The US National Institute of Health Ongoing Trials Register (www.clinicaltrials.gov) and WHO International Clinical Trials Registry Platform (apps.who.int/trialsearch/).

A combination of controlled vocabulary and free-text terms was used for the population (preterm infants) and intervention (surfactant) (see online supplemental eMethods).

10.1136/fetalneonatal-2022-324184.supp1 Supplementary data

### Data collection and management

Two authors independently screened titles and abstracts and selected articles for inclusion based on full-text examination. Two authors independently extracted data in a prepiloted form, including outcome data, data on potential effect modifiers and individual study data (see online supplemental eMethods).

We collected data at maximum follow-up and shorter follow-up where applicable. Trial authors were contacted in the case of missing information. Differences were resolved by discussion. The Cochrane Risk of Bias V.2 tool was used.^16^ Each domain was classified as ‘low risk’, ‘some concern’ or ‘high risk’, leading to classification of the study.

### Measurement of treatment effects

For dichotomous variables the OR with 95% credible intervals (CrI) were calculated.^17^ For continuous variables, we calculated the mean difference with 95% CrI. For count outcomes, we calculated the rate ratio with 95% CrI. For time-to-event outcomes, HR with 95% CI was calculated.

We estimated the ranking probabilities for all interventions (level of FiO_2_) of being at each possible rank for each intervention. We obtained the surface under the cumulative ranking curve (cumulative probability), rankogram and relative ranking table with CrI for the ranking probabilities.^18 19^ The unit of analysis was the participant, according to the intervention group to which the participant was randomly assigned.

### Data synthesis

A network meta-analysis was conducted to compare thresholds of FiO_2_ simultaneously for each outcome. Our analysis was based on guidance by the National Institute for Clinical Excellence Decision Support Unit.^19–21^

We obtained a network plot to ensure that the trials were connected by interventions.^19^ We conducted a Bayesian network meta-analysis using the Markov chain Monte Carlo method (for further details, see online supplemental eMethods). We used fixed-effect and random-effect models, reporting the more conservative. We estimated the probability that each intervention ranks at one of the possible positions.

Analysis was carried out using OpenBUGS V.3.2.3 (OpenBUGS Project Management Group, UK).

We assessed inconsistency (statistical evidence of the violation of transitivity assumption) by fitting both an inconsistency model and a consistency model. In the presence of inconsistency, we assessed whether the inconsistency was due to clinical or methodological heterogeneity. We performed direct comparisons using the same technical details.

Subgroup/sensitivity analysis was planned based on (1) trials at low risk of bias compared with trials at high risk of bias, (2) gestational age and (3) current best practice—use of antenatal steroids and NCPAP.

## Results

**Figure 1:**
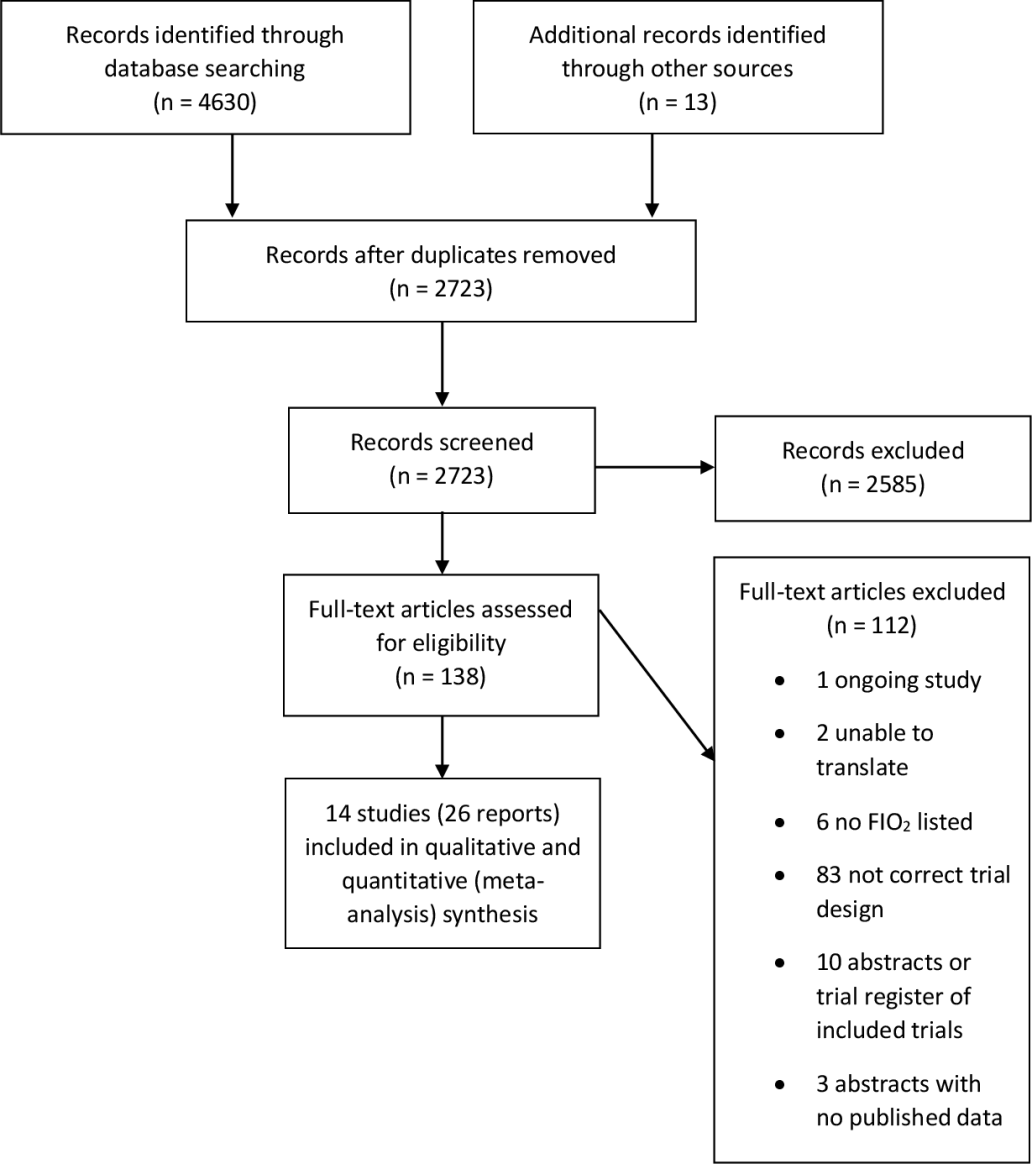
Preferred Reporting Items for Systematic Reviews and Meta-Analyses flow diagram. FiO_2_, fraction of inspired oxygen.

A total of 4643 references were identified. Of 138 full-text articles reviewed, 112 were excluded (see online supplemental eResults). Twenty-six references describing 14 trials were included (PRISMA diagram, figure 1).

The included studies^22–35^ involved 5588 infants, 5298 after postrandomisation dropouts. Threshold of FiO_2_ for provision of selective surfactant ranged from 30% (three studies) to 60% (three studies). Five studies provided surfactants at 40% and three studies provided surfactants at 50%. Mean gestational age ranged from 27 weeks to 30 weeks. The range of gestational ages included in trials was variable as shown in table 1. There does not appear to be a systematic difference in the range of gestational ages among the trials using different FiO_2_ thresholds for selective surfactant provision. Regarding the prophylactic group, in seven studies, surfactant was given straight after birth; in five studies, surfactant was given within 15 min; and in three studies, surfactant was given within 1 hour. The percentage of participants with antenatal steroid exposure ranged from 4% to 99%. Eight studies used Poractant alfa (Curosurf, Chiesi Farmaceutici, Italy). One study allowed Poractant alfa or Beractant (Survanta, AbbVie, USA). Of the five remaining studies, two multicentre trials allowed surfactant as per individual unit protocol, one Calfactant (Infasurf, ONY Biotech, USA) and one a self-prepared bovine surfactant. One study used a self-prepared human surfactant (see table 1 for further details). Twelve publications were identified as follow-up of the cohort in included trials.^36–47^ Due to the nature of the intervention studied, star-shaped networks were formed for each outcome. No closed loops were present, and each study was connected to the network for each outcome. No studies were found to be at low risk of bias, 12 had some concerns; and 2 had high risk of bias (online supplemental eTable 1). As shown in online supplemental eTable 1, there does not appear to be a systematic difference in the risk of bias among the trials using different FiO_2_ thresholds.

**Table 1:** Characteristics of included studies

Study name	Setting	Participants analysed	Threshold for selective surfactant (%)	Primary outcome	Gestational age range (weeks)	Female gender (%)	Antenatal steroids (any) (%)	Surfactant type	Surfactant dose	Ventilation	Dropouts
Kattwinkel *et al* 29	8 centres, USA	1248	30	Moderate RDS*	29–33	47	No info	Bovine Infasurf	150 mg/dose	Both	150
Rojas *et al* 33	8 centres, Columbia	279	30	Need for MV	27–32	49	86	Bovine Survanta	100 mg/kg	CPAP	0
Walti *et al* 35	12 centres, France	256	30	Survival without BPD at 28 days	25–31	46	15	Porcine Curosurf	200 mg/kg	Intubation	32
Bevilacqua *et al* 22	2 centres: Italy and Bulgaria	93	40	Mortality Grade 3, 4 IVH	26–30	54	29	Porcine Curosurf	200 mg/kg	Both	0
Dilmen *et al* 24	6 centres, Turkey	159	40	Necessity for MV	25–30	55	65	Porcine Curosurf	200 mg/kg	CPAP	0
Kendig *et al* 30	3 centres, USA	479	40	Survival to discharge	<30	45	31	Bovine Self-prepared	90 mg/dose	Intubation	0
Lefort *et al* 31	1 centre, Brazil	75	40	Ventilatory parameters	<34	45	No info	Porcine Curosurf	100 mg/kg	Both	0
Sandri *et al* 34	Multicentre, Europe	208	40	MV in first 5 days	25–29	47	97	Porcine Curosurf	200 mg/kg	CPAP	0
Finer *et al* 27	Multicentre, USA	1316	50	Death/BPD at 36 weeks CGA	24–28	46	96	Individual unit protocol	Unit protocol	CPAP	0
Kandraju *et al* 28	1 centre, India	153	50	Need for MV in first week of life	28–34	49	94	Porcine (Curosurf) or bovine (Survanta)	100 mg/kg	CPAP	0
Merritt *et al* 32	3 centres, USA and Finland	148	50	Mortality BPD	24–29	43	4	Human Self-prepared	70 mg/kg	Intubation	98†
de Winter *et al* 23	2 centres, Holland	81	60	TcPO_2_ and FiO_2_ at 6 hrs	26–30	48	44	Porcine Curosurf	200 mg/kg	Intubated	0
Dunn *et al* 25	27 centres: USA and Canada	656	60	Death/BPD at 36 weeks CGA	26–30	49	99	Individual unit protocol	Unit protocol	Both	8
Egberts *et al* 26	4 centres: Sweden and Holland	147	60%	TcPO_2_ and FiO_2_ at 6 hours	26–30	60	29	Porcine Curosurf	200 mg/kg	Intubated	2

*Moderate RDS defined as mean airway pressure ≥8 cmH_2_O or FiO_2_ ≥40%.

†Including 52 patients in placebo group not included in this analysis.

BPD, bronchopulmonary dysplasia; CGA, corrected gestational age; CPAP, continuous positive airway pressure; FiO_2_, fraction of inspired oxygen; IVH, Intraventricular haemorrhage; MV, mechanical ventilation; RDS, respiratory distress syndrome; TcPO_2_, transcutaneous oxygen tension.

### Primary outcome

Each of the 14 studies measured mortality, including 5298 patients. A random-effect model was used. OR for each comparison, Deviance Information Criteria (DIC), median between-study SD and variance are summarised in online supplemental eTable 2. None of the estimates reached statistical significance with 30% threshold having the highest OR for this outcome (1.81) with 95% CrI of 1.0 to 3.44 (table 2). Sensitivity analysis of current best practice (NCPAP use with high rates of antenatal steroid) did not show any statistically significant difference (online supplemental eTables 3 and 4).

**Table 2:** Summary of findings table for the primary outcome mortality at maximal follow-up

Mortality	30% Threshold		40% Threshold		50% Threshold		60% Threshold	
Studies: 14 Participants: 5290								
Prophylaxis: 123 per 1000 (12.3%)	OR 1.81 (1.00 to 3.44) Network estimate	79 more per 1000 (0 fewer to 202 more)	OR 1.52 (0.94 to 2.40) Network estimate	53 more per 1000 (7 fewer to 128 more)	OR 0.82 (0.50 to 1.41) Network estimate	20 fewer per 1000 (57 fewer to 42 more)	OR 1.16 (0.63 to 2.29) Network estimate	17 more per 1000 (41 fewer to 120 more)
Quality of evidence: ⊕⊕◯◯ Low *†	Based on 1783 participants (3 RCTs)		Based on 1014 participants (5 RCTs)		Based on 1617 participants (3 RCTs)		Based on 876 participants (3 RCTs)	

*The trials all had some concerns or were at high risk of bias.

†There was significant heterogeneity.

‡This is a surrogate outcome or was an indirect comparison.

§Less than 300 events in combined groups.

¶There is evidence of publication bias.

RCT, randomised controlled trial.

### Secondary outcomes

ORs, DIC and variance for each comparison can be found in online supplemental eTable 5. A summary of results is provided (tables 3–5).

**Table 3:** Summary of findings table for secondary outcomes: respiratory outcomes

	Threshold 30%		Threshold 40%		Threshold 50%		Threshold 60%	
Bronchopulmonary dysplasia Studies: 8 Participants: 3003								
Prophylaxis: 113 per 1000 (11.3%)	OR 1.39 (0.87 to 2.24) Network estimate	38 more per 1000 (13 fewer to 109 more)	OR 0.77 (0.37 to 1.58) Network estimate	24 fewer per 1000 (68 fewer to 55 more)	OR 0.93 (0.74 to 1.16) Network estimate	7 fewer per 1000 (27 fewer to 16 more)	OR 1.02 (0.72 to 1.45) Network estimate	2 more per 1000 (30 fewer to 43 more)
Quality of evidence: ⊕◯◯◯ Very low *†‡	Based on 279 participants (1 RCT)		Based on 460 participants (3 RCTs)		Based on 1469 participants (2 RCTs)		Based on 795 participants (2 RCTs)	
Chronic lung disease Studies: 9 Participants: 2740								
Prophylaxis: 284 per 1000 (28.4%)	OR 1.48 (0.82 to 2.63) Network estimate	86 more per 1000 (40 fewer to 227 more)	OR 1.05 (0.63 to 1.64) Network estimate	10 more per 1000 (84 fewer to 110 more)	OR 4.08 (0.77 to 35.45) Network estimate	334 more per 1000 (50 fewer to 650 more)	OR 0.59 (0.28 to 1.22) Network estimate	94 fewer per 1000 (185 fewer to 42 more)
Quality of evidence ⊕◯◯◯ Very low *†‡	Based on 1504 participants (2 RCTs)		Based on 855 participants (4 RCTs)		Based on 153 participants (1 RCT)		Based on 228 participants (2 RCTs)	
BPD or CLD Studies: 13 Participants: 5142								
Prophylaxis: 171 per 1000 (17.1%)	OR 1.45 (0.95 to 2.21) Network estimate	59 more per 1000 (7 fewer to 142 more)	OR 0.91 (0.54 to 1.41) Network estimate	13 fewer per 1000 (71 fewer to 54 more)	OR 0.96 (0.59 to 2.00) Network estimate	6 fewer per 1000 (63 fewer to 121 more)	OR 0.86 (0.47 to 1.34) Network estimate	21 fewer per 1000 (83 fewer to 45 more)
Quality of evidence ⊕◯◯◯ Very low *†‡	Based on 1783 participants (3 RCTs)		Based on 1014 participants (5 RCTs)		Based on 1469 participants (2 RCTs)		Based on 876 participants (3 RCTs)	
Pneumothorax Studies: 14 Participants: 5290								
Prophylaxis 33 per 1000 (3.3%)	OR 2.41 (0.61 to 10.48) Network estimate	43 more per 1000 (13 fewer to 232 more)	OR 1.26 (0.42 to 3.97) Network estimate	8 more per 1000 (19 fewer to 87 more)	OR 0.81 (0.19 to 3.47) Network estimate	6 fewer per 1000 (27 fewer to 74 more)	OR 2.05 (0.50 to 10.72) Network estimate	33 more per 1000 (16 fewer to 237 more)
Quality of evidence ⊕◯◯◯ Very low *†§	Based on 1783 participants (3 RCTs)		Based on 1014 participants (5 RCTs)		Based on 1617 participants (3 RCTs)		Based on 876 participants (3 RCTs)	

All results are reported as OR with 95% credible intervals.

*The trials were all had some concerns or were at high risk of bias.

†There was significant heterogeneity.

‡This is a surrogate outcome or was an indirect comparison.

§Less than 300 events in combined groups.

RCT, randomised controlled trial.

**Table 4:** Summary of findings table for secondary outcomes: number of surfactant doses required

Surfactant: doses (n)	Threshold 60%		Threshold 30%		Threshold 50%		Threshold 40%	
Studies: 13 Participants: 5142								
Prophylaxis: 1107 per 1000 (110.7 per 100 participants)	RaR 0.26 (0.21 to 0.32) Network estimate	815 fewer per 1000 (870 fewer to 750 fewer)	RaR 0.51 (0.46 to 0.56) Network estimate	546 fewer per 1000 (602 fewer to 484 fewer)	RaR 0.65 (0.58 to 0.73) Network estimate	384 fewer per 1000 (463 fewer to 297 fewer)	RaR 0.71 (0.63 to 0.81) Network estimate	316 fewer per 1000 (406 fewer to 215 fewer)
Rank: 5 (5–5)	Rank: 1 (1–1)		Rank: 2 (2–2)		Rank: 3 (3–4)		Rank: 4 (3–4)	
Quality of evidence: ⊕⊕◯◯ Low *†	Based on 334 participants (3 RCTs)		Based on 881 participants (3 RCTs)		Based on 742 participants (2 RCTs)		Based on 511 participants (5 RCTs)	

All results are reported as OR with 95% credible intervals.

*The trials were all had some concerns or were at high risk of bias.

†There was significant heterogeneity.

RaR, rate ratio; RCT, randomised controlled trial.

**Table 5:** Summary of findings table for secondary outcome: major morbidities

	30% Threshold		40% Threshold		50% Threshold		60% Threshold	
Total major morbidities (n) Studies: 12 Participants: 5134								
Prophylaxis: 316 per 1000 (31.6 per 100 participants)	RaR 1.14 (0.94 to 1.40) Network estimate	45 more per 1000 (20 fewer to 126 more)	RaR 1.18 (0.89 to 1.56) Network estimate	56 more per 1000 (34 fewer to 176 more)	RaR 1.04 (0.92 to 1.18) Network estimate	14 more per 1000 (25 fewer to 58 more)	RaR 1.02 (0.81 to 1.28) Network estimate	six more per 1000 (62 fewer to 89 more)
Quality of evidence ⊕◯◯◯ Very low*†§	Based on 1783 participants (3 RCTs)		Based on 939 participants (4 RCTs)		Based on 1617 participants (3 RCTs)		Based on 795 participants (2 RCTs)	
Grade 3/4 intraventricular haemorrhage Studies: 12 Participants: 5134								
Prophylaxis 44 per 1000 (4.4%)	OR 2.01 (0.83 to 5.46) Network estimate	40 more per 1000 (7 fewer to 156 more)	OR 1.69 (0.77 to 4.10) Network estimate	28 more per 1000 (10 fewer to 114 more)	OR 1.11 (0.44 to 2.47) Network estimate	5 more per 1000 (24 fewer to 58 more)	OR 0.68 (0.22 to 2.03) Network estimate	14 fewer per 1000 (34 fewer to 41 more)
Quality of Evidence ⊕⊕◯◯ Low *†	Based on 1783 participants (3 RCTs)		Based on 939 participants (4 RCTs)		Based on 1617 participants (3 RCTs)		Based on 795 participants (2 RCTs)	
Periventricular leucomalacia Studies: 8 Participants: 3087								
Prophylaxis: 34 per 1000 (3.4%)	OR 0.81 (0.51 to 1.28) Network estimate	6 fewer per 1000 (16 fewer to nine more)	OR 0.64 (0.07 to 4.25) Network estimate	12 fewer per 1000 (31 fewer to 96 more)	OR 0.80 (0.21 to 2.81) Network estimate	7 fewer per 1000 (27 fewer to 56 more)	OR 0.58 (0.19 to 1.50) Network estimate	14 fewer per 1000 (27 fewer to 16 more)
Quality of evidence: ⊕◯◯◯ Very low *†§	Based on 1783 participants (3 RCTs)		Based on 208 participants (1 RCT)		Based on 301 participants (2 RCTs)		Based on 795 participants (2 RCTs)	
Necrotising enterocolitis Studies: 10 Participants: 4690								
Prophylaxis: 75 per 1000 (7.5%)	OR 0.86 (0.55 to 1.35) Network estimate	10 fewer per 1000 (32 fewer to 24 more)	OR 1.27 (0.81 to 2.01) Network estimate	18 more per 1000 (13 fewer to 65 more)	OR 1.27 (0.91 to 1.77) Network estimate	18 more per 1000 (6 fewer to 51 more)	OR 1.15 (0.61 to 2.10) Network estimate	10 more per 1000 (28 fewer to 70 more)
Quality of evidence: ⊕⊕◯◯ Low *†	Based on 1504 participants (2 RCTs)		Based on 921 participants (4 RCTs)		Based on 1617 participants (3 RCTs)		Based on 648 participants (1 RCT)	
Retinopathy of prematurity >stage 2 Studies: 6 Participants: 3727								
Prophylaxis 52 per 1000 (5.2%)	OR 1.01 (0.01 to 96.83) Network estimate	1 more per 1000 (52 fewer to 790 more)	OR 0.87 (0.09 to 7.05) Network estimate	6 fewer per 1000 (47 fewer to 228 more)	OR 0.99 (0.12 to 6.96) Network estimate	0 fewer per 1000 (45 fewer to 225 more)	OR 2.36 (0.13 to 40.29) Network estimate	63 more per 1000 (45 fewer to 638 more)
Quality of evidence: ⊕◯◯◯ Very low *†§	Based on 1248 participants (1 RCT)		Based on 367 participants (2 RCTs)		Based on 1464 participants (2 RCTs)		Based on 648 participants (1 RCT)	
BPD Studies: 8 Participants: 3003								
Prophylaxis: 113 per 1000 (11.3%)	OR 1.39 (0.87 to 2.24) Network estimate	38 more per 1000 (13 fewer to 109 more)	OR 0.77 (0.37 to 1.58) Network estimate	24 fewer per 1000 (68 fewer to 55 more)	OR 0.93 (0.74 to 1.16) Network estimate	7 fewer per 1000 (27 fewer to 16 more)	OR 1.02 (0.72 to 1.45) Network estimate	2 more per 1000 (30 fewer to 43 more)
Quality of evidence: ⊕◯◯◯ Very low*†‡	Based on 279 participants (1 RCT)		Based on 460 participants (3 RCT)		Based on 1469 participants (2 RCT)		Based on 795 participants (2 RCT)	

All results are reported as OR with 95% credible intervals.

*The trials were all had some concerns or were at high risk of bias.

†There was significant heterogeneity.

‡This is a surrogate outcome or was an indirect comparison.

§Less than 300 events in combined groups.

¶There is evidence of publication bias.

BPD, bronchopulmonary dysplasia; RaR, rate ratio; RCT, randomised controlled trial.

#### Respiratory outcomes

BPD, CLD and CLD/BPD at maximum follow-up were assessed. There was no difference regarding BPD or CLD alone. When evaluated at maximum follow-up, incidence was higher in the 30% group than prophylaxis when directly compared. The other outcomes showed lower point estimates, although not reaching statistical significance.

### Use of surfactant

Unsurprisingly, the proportion of infants receiving surfactant was significantly higher in the prophylactic group (online supplemental eTable 5e).

Regarding the number of surfactant doses, there was a significant difference between thresholds. The 60% threshold had the least use of surfactant, 815 fewer doses per 1000. The 30% threshold ranked second at 546 fewer doses per 1000; the 50% threshold ranked third at 384 fewer doses per 1000; and the 40% threshold ranked last at 316 fewer doses per 1000.

### Complications of prematurity

We showed no significant differences in incidence of IVH, PVL, NEC or BPD. The 60% threshold showed a higher incidence of ROP on direct comparison with prophylaxis (OR 2.35, 95% CrI 1.02 to 5.42). Due to the presentation of components of this outcome separately in included studies, we performed a combined count outcome. Studies were included if they provided data from two or more of the five components of the composite outcome. No significant differences were found.

#### Neurodevelopment at CGA of 2 years

One trial^27^ reported this outcome. Forty-three of 479 in the prophylactic group and 55 of 511 in the selective group developed one or more component.

#### Health-related quality of life

No study assessed HRQOL.

### Quality of evidence

The overall quality of the evidence was low or very low for all comparisons due to the high risk of bias, heterogeneity, indirectness, imprecision and publication bias.

### Heterogeneity

Since there was no meaningful way in which to rank these studies, we were unable to perform the comparison-adjusted funnel plot to assess reporting bias. Due to paucity of data, we were unable to perform planned subgroup analyses based on gestation, type of ventilation or antenatal steroid use alone. To explore heterogeneity, a sensitivity analysis was carried out comparing studies using current best practice (over 60% antenatal steroid use and NCPAP for stabilisation).

#### NCPAP and high antenatal steroid use

A summary of findings is shown in table 6. Six studies^24 25 27 28 33 34^ met the criteria, including 2554 infants. There was no statistically significant difference seen in mortality, BPD, pneumothorax or grade 3/4 IVH. There was an increased rate of major morbidity in the 60% threshold group—310 more per 1000 (95% CrI intervals 136 more to 572 more). ORs, DIC and variance for each comparison are provided in online supplemental eTables 3 and 4. Each comparison had a very low quality of evidence.

**Table 6:** Sensitivity analysis of current best practice (stabilisation with NCPAP and high levels of antenatal steroid use)

	30% Threshold		40% Threshold		50% Threshold		60% Threshold	
Mortality								
Prophylaxis: 103 per 1000 (10.3%)	OR 1.03 (0.45 to 2.35) Network estimate	2 more per 1000 (54 fewer to 110 more)	OR 1.32 (0.69 to 2.61) Network estimate	29 more per 1000 (29 fewer to 127 more)	OR 0.81 (0.61 to 1.07) Network estimate	18 fewer per 1000 (38 fewer to seven more)	OR 0.56 (0.23 to 1.29) Network estimate	43 fewer per 1000 (78 fewer to 26 more)
Quality of evidence: ⊕◯◯◯ Very low *†‡§	Based on 279 participants (1 RCT)		Based on 367 participants (2 RCTs)		Based on 1469 participants (2 RCTs)		Based on 439 participants (1 RCT)	
Bronchopulmonary dysplasia								
Prophylaxis: 175 per 1000 (17.5%)	OR 1.40 (0.88 to 2.24) Network estimate	54 more per 1000 (18 fewer to 148 more)	OR 0.83 (0.39 to 1.70) Network estimate	26 fewer per 1000 (99 fewer to 91 more)	OR 0.93 (0.74 to 1.16) Network estimate	11 fewer per 1000 (39 fewer to 22 more)	OR 1.29 (0.84 to 2.02) Network estimate	41 more per 1000 (25 fewer to 125 more)
Quality of evidence: ⊕◯◯◯ Very low*†‡	Based on 279 participants (1 RCT)		Based on 367 participants (2 RCT)		Based on 1469 participants (2 RCT)		Based on 439 participants (1 RCT)	
Pneumothorax								
Prophylaxis: 27 per 1000 (2.7%)	OR 4.99 (0.00 to 6953.50) Network estimate	94 more per 1000 (27 fewer to 968 more)	OR 3.09 (0.02 to 2455.29) Network estimate	52 more per 1000 (26 fewer to 959 more)	OR 1.52 (0.01 to 324.08) Network estimate	14 more per 1000 (27 fewer to 873 more)	OR 1.73 (0.00 to 2151.67) Network estimate	19 more per 1000 (27 fewer to 957 more)
Quality of evidence: ⊕◯◯◯ Very low*†‡§	Based on 279 participants (1 RCT)		Based on 367 participants (2 RCTs)		Based on 1469 participants (2 RCTs)		Based on 439 participants (1 RCT)	
Major morbidity								
Prophylaxis: 296 per 1000 (29.6 per 100 participants)	RaR 1.20 (0.86 to 1.68) Network estimate	60 more per 1000 (41 fewer to 202 more)	RaR 1.16 (0.81 to 1.66) Network estimate	47 more per 1000 (56 fewer to 196 more)	RaR 1.06 (0.93 to 1.21) Network estimate	19 more per 1000 (20 fewer to 62 more)	RaR 2.05 (1.46 to 2.93) Network estimate	310 more per 1000 (136 more to 572 more)
Quality of evidence: ⊕◯◯◯ Very low*†‡§	Based on 279 participants (1 RCT)		Based on 367 participants (2 RCTs)		Based on 1469 participants (2 RCTs)		Based on 439 participants (1 RCT)	
Grade 3/4 intraventricular haemorrhage								
Prophylaxis: 39 per 1000 (3.9%)	OR 1.64 (0.24 to 14.41) Network estimate		23 more per 1000 (29 fewer to 329 more)		OR 2.16 (0.87 to 5.98) Network estimate		41 more per 1000 (5 fewer to 156 more)	
Quality of evidence: ⊕◯◯◯ Very low*†‡§	Based on 279 participants (1 RCT)		Based on 367 participants (2 RCTs)		Based on 1469 participants (2 RCTs)		Based on 439 participants (1 RCT)	

*The trials were all had some concerns or were at high risk of bias.

†There was significant heterogeneity.

‡This is a surrogate outcome or was an indirect comparison.

§Less than 300 events in combined groups.

¶There is evidence of publication bias.

NCPAP, nasal continuous positive airway pressure; RaR, rate ratio; RCT, randomised conrolled trial.

## Discussion

Our primary outcome, mortality, showed no statistically significant differences between the thresholds of FiO_2_ examined. Regarding the major morbidities of preterm birth, the 60% threshold showed a higher incidence of ROP on direct comparison with prophylaxis. Regarding surfactant doses received, there was significant differences between thresholds. The 60% threshold had the least doses, 30% threshold second, 50% threshold third and 40% threshold last. This may suggest that earlier selective treatment decreases the need for repeat doses, and that earlier use of surfactant may be appropriate as infants reaching this threshold will need more surfactant if treatment is delayed. However, this would be contradicted by the 60% threshold requiring least doses. Interpretation is complicated by differences in rescue dosing, dosing strategies between studies and total amount of doses allowed. The 30% threshold, despite having less doses of surfactant, had a higher incidence of prolonged respiratory support. This may relate to exposure to harmful effects of ventilation earlier, when the neonatal lung is more vulnerable.

A sensitivity analysis of infants treated with the current standard of care showed an increase in major morbidity in the 60% threshold group. While our analysis failed to identify an optimal threshold, it adds to scarce data. In the absence of evidence showing a benefit to treatment at 30%, 40% or 50% FiO_2_, it warrants consideration of higher thresholds (except 60%)—decreasing invasive procedures, associated mechanical ventilation, surfactant use, sedation and associated side effects. The economic impact is likely to be significant.

Despite the common nature of this issue, there are little data to guide clinicians. A secondary analysis of prospectively collected data^6^ has been used to support lower thresholds. This study reviewed infants between 25 weeks and 32 weeks of gestation initially managed on NCPAP. Multivariate analysis showed NCPAP failure was predicted by the highest FiO_2_ in the first hours. This study was limited by several factors: its retrospective nature, the small numbers at each gestation and the low number primarily managed with NCPAP (50%). The authors concluded that NCPAP failure was predicted by an FiO_2_ greater than 30% in the first hours and was associated with adverse outcomes. A review of the literature by Dani^5^ also evaluated this issue, concluding that the most effective threshold is unknown.

The European Consensus Guidelines on the management of RDS,^2^ based on the above paper by Dargaville *et al*,^6^ suggests ‘early’ use of rescue surfactant outside of the delivery room at an FiO_2_ of 30% or above. However, the guideline also recommends using 30%–40% FiO_2_ for initial stabilisation despite advising against prophylactic surfactant.

Despite the common use of FiO_2_ as a major criterion for provision of selective surfactant, there are limitations to its use, especially in isolation. A combination of pH, clinical assessment and FiO_2_ will give a more accurate assessment. FiO_2_ can be influenced by many factors including NCPAP interface, mode of non-invasive ventilation and level of positive end expiratory pressure and can be a measure of pathologies other than surfactant deficiency.

The strength of this review was the range of databases searched without restrictions. Two independent reviewers carried out article identification and data extraction. Analysis was performed using fixed-effect and random-effect models, with the most conservative reported. There were limitations. A scoping search revealed no studies directly comparing thresholds for provision of surfactant, and therefore, we relied on indirect comparisons. A paucity of data decreased confidence in results and precluded planned analyses.

There was a lack of long-term neurodevelopmental follow-up and assessment of quality of life. As survival rates of prematurity increase, long-term effects become increasingly important. Parental perspective is vital in this regard.

## Conclusion

This network meta-analysis of 14 studies and 5290 infants suggests no statistically significant difference between a range of 30% to 50% FiO_2_ for the provision of surfactant to preterm infants regarding mortality, respiratory outcomes or complications of prematurity. A 60% threshold may result in more major morbidities. Despite the low quality of evidence and limitations of indirect comparisons, this review provides the strongest evidence currently available, supporting more judicious use of surfactant in preterm infants.

## Data Availability

Data are available upon reasonable request. Data is available on reasonable request to the authors.
