# Supplementary material for: Thresholds for surfactant use in preterm neonates: a network meta-analysis
Source: Arch Dis Child Fetal Neonatal Ed. 2022 Dec 9;108(4):333–41. doi: 10.1136/archdischild-2022-324184 (PMC10313962; doi:10.1136/archdischild-2022-324184)
Supplement: Supplementary data [file fetalneonatal-2022-324184supp001.pdf]

BMJ Publishing Group Limited (BMJ) disclaims all liability and responsibility arising from any reliance placed on this supplemental material which has been supplied by the author(s)

Online Only Material

1) eMethods - Search Strategy
2) eMethods - Data extraction and Management
3) eMethods - Data Synthesis
4) eResults - Risk of Bias Assessment
   eTable 1 – Risk of Bias Assessment
5) eResults - Excluded Studies.
6) eResults - Primary Outcome Mortality
   eTable 2 – Odds ratio for both the direct and network comparison of the primary outcome
7) eResults - Secondary Outcomes
   eTable 3 - Odds ratio for both the direct and network comparison for secondary outcomes
8) eResults - Sensitivity Analysis of Current Best Practice
   eTable 4 - Odds Ratio for Sensitivity Analysis
   eTable 5 - Models used for outcomes for sensitivity analysis

eMethods – Search Strategy

1) **The Central Register of Controlled Trials (CENTRAL) in the Cochrane Library**
Searched from inception to March 2020
Search strategy:

1 MeSH descriptor: [Infant, Very Low Birth Weight] explode all trees
#2 MeSH descriptor: [Infant, Newborn] explode all trees
#3 MeSH descriptor: [Infant, Extremely Low Birth Weight] explode all trees
#4 MeSH descriptor: [Infant, Low Birth Weight] explode all trees
#5 MeSH descriptor: [Infant, Premature] explode all trees
#6 MeSH descriptor: [Infant, Extremely Premature] explode all trees
#7 MeSH descriptor: [Premature Birth] explode all trees
#8 #1 OR #2 OR #3 OR #4 OR #5 OR #6 OR #7
#9 Birth, Premature OR Births, Premature OR Premature Births OR Preterm Birth OR Birth, Preterm OR Births, Preterm OR Preterm Births OR Infants, Premature OR Premature Infant OR Preterm Infants OR Infant, Preterm OR Infants, Preterm OR Preterm Infant OR Premature Infants OR Neonatal OR Prematurity OR Prematurity, Neonatal OR Extremely Premature Infant OR Infants, Extremely Premature OR Premature Infant, Extremely OR Premature Infants, Extremely OR Extremely Preterm Infants OR Extremely Preterm Infant OR Infant, Extremely Preterm OR Infants, Extremely Preterm OR Preterm Infant, Extremely OR Preterm Infants, Extremely OR Extremely Premature Infants OR Extremely Low Birth Weight OR Extremely Low Birth Weight Infant OR (Extremely AND low AND birth AND weight) OR Very Low Birth Weight OR (Very AND low AND birth AND weight) OR Very Low Birth Weight Infant OR Low Birth Weight Infant OR Low Birth Weight OR Birth Weight, Low OR Birth Weights, Low OR Low Birth Weights Infants, Newborn OR Newborn Infant OR Newborn Infants OR Newborns OR Newborn OR Neonate OR Neonates OR Infants, Premature OR Premature Infant OR Preterm Infants OR Infant, Preterm OR Infants, Preterm OR Preterm Infant OR Premature Infants OR Neonatal Prematurity OR Prematurity, Neonatal OR ELBW OR VLBW OR Extreme prematurity
#10 #8 OR #9
#11 MeSH descriptor: [Surface-Active Agents] explode all trees
#12 MeSH descriptor: [Pulmonary Surfactants] explode all trees
#13 Agents, Surface-Active OR Surface Active Agents OR Active Agents, Surface OR Agents, Surface Active OR Tensides OR Surfactants OR Surfactant OR Amphiphilic Agents OR Agents, Amphiphilic OR Surfactants, Pulmonary OR Pulmonary Surfactant OR Surfactant, Pulmonary
#14 #11 OR #12 OR #13
#15 #8 AND #14

2) **MEDLINE (PubMed)**
Searched from inception to March 2020
Search strategy:

a) Population – neonates
"Infant, Newborn"[Mesh]
"Infant, Extremely Low Birth Weight"[Mesh]
"Infant, Low Birth Weight"[Mesh]
"Infant, Very Low Birth Weight"[Mesh]
"Infant, Premature"[Mesh]
“Extremely Premature"[Mesh]
"Premature Birth"[Mesh]

Entry terms:
Birth, Premature
Births, Premature

Premature Births
Preterm Birth
Birth, Preterm
Births, Preterm
Preterm Births
Infants, Premature
Premature Infant
Preterm Infants
Infant, Preterm
Infants, Preterm
Preterm Infant
Premature Infants
Neonatal Prematurity
Prematurity, Neonatal
Extremely Premature Infant
Infants, Extremely Premature
Premature Infant, Extremely
Premature Infants, Extremely
Extremely Preterm Infants
Extremely Preterm Infant
Infant, Extremely Preterm
Infants, Extremely Preterm
Preterm Infant, Extremely
Preterm Infants, Extremely
Extremely Premature Infants
Extremely-Low-Birth-Weight [all fields] OR
Extremely Low Birth Weight [all fields] OR
Extremely Low Birth Weight Infant
Extremely AND low AND birth AND weight [all fields] OR
Very Low Birth Weight [all fields] OR
Very Low-Birth-Weight [all fields] OR
Very AND low AND birth AND weight OR
Infant, Very-Low-Birth-Weight [all fields] OR
Infants, Very-Low-Birth-Weight [all fields] OR
Very Low Birth Weight Infant [all fields] OR
Very-Low-Birth-Weight Infants [all fields] OR
Low-Birth-Weight Infant
Infant, Low-Birth-Weight
Infants, Low-Birth-Weight
Low Birth Weight Infant
Low-Birth-Weight Infants
Low Birth Weight
Birth Weight, Low
Birth Weights, Low
Low Birth Weights
Infants, Newborn [all fields] OR
Newborn Infant [all fields] OR
Newborn Infants [all fields] OR
Newborns [all fields] OR
Newborn [all fields] OR
Neonate [all fields] OR
Neonates [all fields] OR
ELBW [all fields] OR
VLBW [all fields] OR
Extreme prematurity [all fields]

b) <u>Intervention – Surfactant</u>
34 "Surface-Active Agents"[Mesh]
35 "Pulmonary Surfactants"[Mesh]

Entry terms
36 Agents, Surface-Active [all fields] OR
37 Surface Active Agents [all fields] OR
38 Active Agents, Surface [all fields] OR
39 Agents, Surface Active [all fields] OR
40 Tensides [all fields] OR
41 Surfactants [all fields] OR
42 Surfactant [all fields] OR
43 Amphiphilic Agents [all fields] OR
44 Agents, Amphiphilic [all fields] OR
45 Surfactants, Pulmonary [all fields] OR
46 Pulmonary Surfactant [all fields] OR
47 Surfactant, Pulmonary [all fields]

The population and intervention search strategies above were combined with boolen operator 'AND'. The pubmed controlled clinical trials filter was applied.

3) EMBASE

Searched from inception to March 2020
Search strategy:

1. infant newborn.mp. or exp newborn/
2. extremely low birth weight.mp. or exp low birth weight/ or exp very low birth weight/ or exp extremely low birth weight/ or exp newborn/ or exp prematurity/
3. extremely-low-birth-weight.mp.
4. (extremely and low and birth and weight).mp. [mp=title, abstract, heading word, drug trade name, original title, device manufacturer, drug manufacturer, device trade name, keyword, floating subheading word, candidate term word]
5. very low birth weight.mp. or exp very low birth weight/
6. very-low-birth-weight.mp.
7. (very and low and birth and weight).mp. [mp=title, abstract, heading word, drug trade name, original title, device manufacturer, drug manufacturer, device trade name, keyword, floating subheading word, candidate term word]
8. newborn infant.mp.
9. neonate.mp.
10. premature.mp.
11. exp premature labor/ or preterm.mp. or exp gestational age/
12. elbw.mp.
13. vlbw.mp.
14. 1 or 2 or 3 or 4 or 5 or 6 or 7 or 8 or 9 or 10 or 11 or 12 or 13
15. exp surfactant associated protein/ or exp surfactant/ or surfactant.mp.
16. pulmonary surfactant.mp. or exp lung surfactant/
17. surface active agents.mp. or surfactant/
18. surfactant/ or tensides.mp.
19. surfactants.mp. or surfactant/
20. 15 or 16 or 17 or 18 or 19
21. (random* or factorial* or crossover* or cross over* or cross-over* or placebo* or doubl* blind* or singl* blind* or assign* or allocat* or volunteer*).af.
22. exp crossover-procedure/ or exp double-blind procedure/ or exp randomized controlled trial/ or exp single-blind procedure/
23. 21 or 22
24. 14 and 20 and 23

4) **Science Citation Index Expanded (Web of Science)**
Searched from inception to March 2020
Search strategy:

TI = (Surface-Active Agents OR Pulmonary Surfactants OR Agents, Surface-Active OR Active Agents, Surface OR Agents, Surface Active OR Surfactants OR Surfactant OR Surfactants, Pulmonary OR Pulmonary Surfactant OR Surfactant, Pulmonary)

TI = (Infant, Extremely Low Birth Weight OR Infant, Low Birth Weight OR Infant, Very Low Birth Weight OR Infant, Premature OR Extremely Premature)

TI = (Infants, Premature OR Premature Infant OR Preterm Infants OR Infant, Preterm OR Preterm Infant OR Premature Infants OR Neonatal Prematurity OR Prematurity, Neonatal OR Extremely Premature Infant OR Infants, Extremely Premature OR Premature Infant, Extremely OR Premature Infants, Extremely OR Extremely Preterm Infants)

TI = (Extremely Preterm Infant OR Extremely Premature Infants OR Extremely-Low-Birth-Weight OR Extremely Low Birth Weight [all fields] OR(Extremely AND low AND birth AND weight) OR Very Low Birth Weight OR Very Low-Birth-Weight OR Neonate OR Neonates OR ELBW OR VLBW OR extreme prematurity)

TS=(random* OR rct* OR crossover OR masked OR blind* OR placebo* OR meta-analysis OR systematic review* OR meta-analys*)

5) **ClinicalTrials.gov**
Searched from inception to March 2020
Condition: prematurity
Intervention: Surfactant

6) **World Health Organization International Clinical Trials Registry Platform (apps.who.int/trialsearch/)**
Searched from inception to March 2020
Condition: neo*
Intervention: surfactant

eMethod – Data extraction and Management

Two authors independently extracted the data below in a pre-piloted data extraction form:

- Outcome data (for each outcome and each intervention group):
  - Number of participants randomised
  - Number of participants included for the analysis
  - Number of participants with events for binary outcomes, mean and standard deviation for continuous outcomes, number of events and the mean follow-up period for count outcomes and number of participants with events and the mean follow-up period for time-to-event outcomes
  - Natural logarithm of hazard ratio and its standard error if this was reported rather than the number of participants with events and the mean follow-up period for time-to-event outcomes
- Data on potential effect modifiers:
  - Participant characteristics such as sex, gestational age, birthweight, use of antenatal steroids
  - Details of the intervention and control
  - Length of follow-up
  - Information related to 'Risk of Bias' assessment
- Other data:
  - Year and language of publication
  - Country
  - Year(s) in which the trial was conducted
  - Inclusion and exclusion criteria

We collected data at maximum follow-up provided and also at shorter (up to three months) and medium-term follow-up (three months to 1 year) where applicable. We attempted to contact trial authors in the case of unclear or missing information. Any differences in opinion were resolved by discussion.

eMethods – Data Synthesis

A network meta-analysis was conducted to compare thresholds of FiO2 simultaneously for each of the primary and secondary outcomes. Network meta-analysis combines direct evidence within trials and indirect evidence across trials [1]. Our analysis was based on guidance by the National Institute for Clinical Excellence (NICE) Decision Support Unit (DSU).[1-4]

We obtained a network plot to ensure that the trials were connected by interventions [3]. We excluded any trials unconnected to the network from the meta-analysis and reported only the direct pair-wise meta-analysis for such comparisons.

We conducted a Bayesian network meta-analysis using the Markov chain Monte Carlo method.

We used a fixed-effect model and random-effects model for the network meta-analysis. For each pair-wise comparison in a table, we reported the fixed-effect model if the two models reported similar results; otherwise, we reported the more conservative model.

We used a hierarchical Bayesian model using three different initial values, employing codes provided by NICE DSU [5]. We used a normal distribution with large variance (10,000) for treatment effect priors (vague or flat priors). For the random-effects model, we used a prior distributed uniformly (limits: 0 to 5) for between-trial standard deviation but assumed the same between-trial standard deviation across treatment comparisons [5]. We used a 'burn-in' of 10,000 simulations, checked for convergence (of effect estimates and between-study heterogeneity) visually (i.e. whether the values in different chains mix very well by visualisation) and ran the models for another 10,000 simulations to obtain effect estimates. If we did not obtain convergence, we increased the number of simulations for the 'burn-in'.

We estimated the probability that each intervention ranks at one of the possible positions using the NICE DSU codes [5].

Analysis was carried out using OpenBUGS, version 3.2.3

We assessed inconsistency (statistical evidence of the violation of transitivity assumption) by fitting both an inconsistency model and a consistency model. We used inconsistency models employed in the NICE DSU manual, as we used a common between-study standard deviation [2].

In the presence of inconsistency, we assessed whether the inconsistency was due to clinical or methodological heterogeneity.

We performed the direct comparisons using the same codes and the same technical details

Subgroup/sensitivity analysis: Subgroup analysis was planned based on 1) trials at low risk of bias compared to trials at high risk of bias, 2) gestational age, 3) Current best practice – use of antenatal steroids and NCPAP. Due to a paucity of data these could not be carried out. A sensitivity analysis of current best practice was performed. No trials reported only per-protocol analysis results, therefore no best-worst case scenario/worst-best case scenario analyses as sensitivity analyses were required. No imputations were required for mean or standard deviation, therefore sensitivity analysis excluding same was not required.

eResults – Risk of Bias Assessment

eTable 1 – Risk of Bias Assessment

	Bev.	DuW	Dilm	Dunn	Egb	Finer	Kand	Katt	Kend	Lefor	Merr	Roja	Sand	Walti
**Randomisation process**	Low	Low	Low	Low	Low	Low	Low	Low	Low	Low	Low	Low	Low	Low
**Deviations from intended interventions**	Low	SC	High	Low	SC	Low	SC	High	Low	SC	Low	Low	Low	SC
**Missing outcome data**	Low	Low	Low	Low	Low	Low	Low	Low	Low	Low	Low	Low	Low	Low
**Measurement of the outcome**	SC	Low	Low	Low	Low	Low	Low	Low	Low	Low	Low	Low	Low	Low
**Selection of the reported result**	SC	SC	SC	SC	SC	SC	SC	SC	SC	SC	SC	SC	SC	SC
**Overall risk of bias**	SC	SC	High	SC	SC	SC	SC	High	SC	SC	SC	SC	SC	SC

eTable 1a – Risk of bias in each domain for each included study, Author 1. SC some concerns

	Bev.	DuW.	Dilm	Dunn	Egb.	Finer	Kand	Katt.	Kend	Lefor	Merri	Roja	Sand	Walti
**Randomisation process**	Low	Low	Low	Low	Low	Low	Low	Low	Low	Low	Low	Low	Low	Low
**Deviations from intended interventions**	Low	SC	High	Low	SC	Low	SC	High	Low	SC	Low	Low	Low	SC
**Missing outcome data**	Low	Low	Low	Low	Low	Low	Low	Low	Low	Low	Low	Low	Low	Low
**Measurement of the outcome**	Low	Low	Low	Low	Low	Low	Low	Low	Low	Low	Low	Low	Low	Low
**Selection of the reported result**	SC	SC	SC	SC	SC	SC	SC	SC	SC	SC	SC	SC	SC	SC
**Overall risk of bias**	SC	SC	High	SC	SC	SC	SC	SC	SC	SC	SC	SC	SC	SC

eTable 1b – Risk of bias in each domain for each included study, Author 2. SC some concerns

eResults – Excluded Studies

None of the excluded studies met the inclusion criteria.

5 of the studies were identified as review articles or systematic reviews [6-10]. 1 study is an ongoing trial assessing surfactant thresholds for treatment [11]. We were unable to translate 2 studies and the abstracts did not provide sufficient information for inclusion [12,13]. 23 were not randomised control trials [14-36]. 6 trials met the inclusion criteria but did not list an fio2 for treatment with selective surfactant [37-42]. 55 did not meet the inclusion criteria of a trial assessing prophylactic treatment with surfactant vs selective treatment with surfactant [43-96]. 10 of the references are trial register or published abstracts of an included trial: [97-106]. 3 references were abstracts without a published trial found despite attempts to contact the author [107-109].

eResults – Primary Outcome Mortality

A random-effect model was used for the network meta-analysis because it was more conservative.
Deviance Information Criteria (DIC) for fixed model was 171.1, random 172.3.
Median between-study standard deviation for the random-effect model 0.23 (95% CrI 0.011, 0.742), variance 0.055.
Model used for direct comparisons are included in Table 1 with the odds ratio for each comparison.

eTable 2. Odds ratio for both the direct and network comparison of the primary outcome

Mortality	Prophylaxis	Threshold 30%	Threshold 40%	Threshold 50%	Threshold 60%
**Prophylaxis**		1.88[0.25,16.51] *	1.52[0.87,2.52] *	0.8[0.62,1.04] #	1.1[0.67,1.78] #
**Threshold 30%**	1.81[1.00,3.44]		-	-	-
**Threshold 40%**	1.52[0.94,2.40]	0.84[0.37,1.77]		-	-
**Threshold 50%**	0.82[0.50,1.41]	0.45[0.20,1.01]	0.54[0.28,1.13]		-
**Threshold 60%**	1.16[0.63,2.29]	0.64[0.27,1.60]	0.76[0.36,1.80]	1.41[0.64,3.31]	

eTable 2. Odds ratio for both the direct and network comparison of the primary outcome
Network Meta-analysis comparisons are below the greyed line, direct comparisons are above the line. Comparisons that did not reach statistical significance (credibility intervals for the odds ratio cross 1) are highlighted in yellow. Comparisons that do reach statistical significance are highlighted in blue.
Most conservative method of analysis was used in each case. * denotes fixed-effect model, # denotes random effect model for direct comparisons.

BMJ Publishing Group Limited (BMJ) disclaims all liability and responsibility arising from any reliance placed on this supplemental material which has been supplied by the author(s)

eResults – Sensitivity Analysis of Current Best Practice

Six studies met the criteria. This included 2554 patients. 1268 were in the combined prophylaxis arm and were compared with 138 (one study) in the 30% threshold arm, 183 (2 studies) in the 40% arm, 727 (two studies) in the 50% arm and 216 (one study) in the 60% arm.

eTable 4 shows the odds ratio for each comparison within the analysis, along with the model of comparison used. Most conservative model was used in each case.

Fixed-effects model was used for all outcomes, except pneumothorax, where random-effects model was used. DIC, between-study variance with 95% CrI and variance where applicable are shown in eTable 5.

There was no statistically significant difference seen in mortality, BPD, pneumothorax, or grade 3/4 IVH.

There was an increased rate of major morbidity in the 60% threshold group– 31 more per 1000 (95% CrI intervals 136 more to 572 more).

Each comparison was deemed to be at very-low quality of evidence.

eTable 3. Odds Ratio for Sensitivity Analysis

Mortality	Prophylaxis	30% Threshold	40% Threshold	50% Threshold	60% Threshold
Prophylaxis		1.02[0.45,2.34] &	1.33[0.69,2.6] *	0.81[0.61,1.07] *	0.55[0.23,1.29] &
30% Threshold	1.03[0.45,2.35]		-	-	-
40% Threshold	1.32[0.69,2.61]	1.30[0.45,3.77]		-	-
50% Threshold	0.81[0.61,1.07]	0.79[0.33,1.90]	0.61[0.29,1.24]		-
60% Threshold	0.56[0.23,1.29]	0.54[0.16,1.77]	0.42[0.14,1.22]	0.69[0.27,1.66]	

eTable 3a. Odds ratio for sensitivity analysis for mortality

Bronchopulmonary Dysplasia	Prophylaxis	30% Threshold	40% Threshold	50% Threshold	60% Threshold
Prophylaxis		1.39[0.87,2.23]	0.83[0.39,1.7]	0.93[0.74,1.16]	1.3[0.84,2.02]
30% Threshold	1.40[0.88,2.24]		-	-	-
40% Threshold	0.83[0.39,1.70]	0.59[0.24,1.40]		-	-
50% Threshold	0.93[0.74,1.16]	0.66[0.39,1.11]	1.12[0.53,2.44]		-
60% Threshold	1.29[0.84,2.02]	0.93[0.49,1.76]	1.57[0.68,3.74]	1.40[0.86,2.30]	

eTable 3b. Odds ratio for sensitivity analysis for bronchopulmonary dysplasia

Pneumothorax	Prophylaxis	30% Threshold	40% Threshold	50% Threshold	60% Threshold
Prophylaxis		4.78[1.42,22.97] &	3.73[0.01,3209.92]#	1.07[0.71,1.62] *	1.73[0.67,4.82] &
30% Threshold	4.99[0.00,6953.50]		-	-	-
40% Threshold	3.09[0.02,2455.29]	0.65[0.00,14472.42]		-	-
50% Threshold	1.52[0.01,324.08]	0.31[0.00,2426.00]	0.48[0.00,754.46]		-
60% Threshold	1.73[0.00,2151.67]	0.36[0.00,8681.94]	0.54[0.00,2972.03]	1.13[0.00,8391.71]	

eTable 3c. Odds ratio for sensitivity analysis for pneumothorax

Major Morbidity	Prophylaxis	30% Threshold	40% Threshold	50% Threshold	60% Threshold
Prophylaxis		1.21[0.87,1.7] &	1.15[0.8,1.66] *	1.06[0.93,1.21] *	2.05[1.45,2.92] &
30% Threshold	1.20[0.86,1.68]		-	-	-
40% Threshold	1.16[0.81,1.66]	0.96[0.58,1.57]		-	-
50% Threshold	1.06[0.93,1.21]	0.88[0.62,1.26]	0.92[0.63,1.34]		-
60% Threshold	2.05[1.46,2.93]	1.70[1.05,2.78]	1.77[1.07,2.95]	1.92[1.34,2.83]	

eTable 3d. Odds ratio for sensitivity analysis for major morbidity

Grade 3 or 4 Intraventricular Haemorrhage	Prophylaxis	30% Threshold	40% Threshold	50% Threshold	60% Threshold
Prophylaxis		1.62[0.24,14.17] &	2.16[0.86,5.88] *	1.28[0.93,1.78] *	0.71[0.23,2.12] &
30% Threshold	1.64[0.24,14.41]		-	-	-
40% Threshold	2.16[0.87,5.98]	1.32[0.12,11.55]		-	-
50% Threshold	1.28[0.93,1.78]	0.78[0.09,5.46]	0.59[0.21,1.56]		-
60% Threshold	0.71[0.23,2.09]	0.43[0.04,3.90]	0.33[0.07,1.36]	0.55[0.17,1.71]	

eTable 3e. Odds ratio for sensitivity analysis for grade 3 or 4 Intraventricular Haemorrhage

eTable 3 (a-e) above shows the odds ratio for the network and direct comparisons for each outcome in the sensitivity analysis.
Network meta-analysis comparisons are below the greyed line, direct comparisons are above the line.
Comparisons that did not reach statistical significance (credibility intervals for the odds ratio cross 1) are highlighted in yellow. Comparisons that do reach statistical significance are highlighted in blue.
Most conservative model of analysis was used in each case.
*denotes fixed-effect model, # denotes random effect model for the direct comparison, & denotes only one study in comparison leading to use of the random effects model,
^ denotes zero events in at least one arm of one study leading to use of the fixed effect model.

eTable 4. Models used for outcomes for sensitivity analysis

Outcome	DIC – Fixed	DIC - Random	Model Used	SD	95% CrI	Variance
**Mortality**	74.72	76.47	Fixed			
**BPD**	76.01	76.54	Fixed			
**Pneumothorax**	75.86	63.82	Random	3.424	1.22, 4.92	11.72
**Major Morbidity**	89.54	89.58	Fixed			
**Grade 3/4 IVH**	66.33	67.54	Fixed			

eTable 4. Models used for outcomes for sensitivity analysis
DIC – Deviance Information Criteria, Fixed – Fixed effect model, Random – Random effect model
SD – between study standard deviation, CrI – Credible interval, BPD – bronchopulmonary dysplasia, IVH – intraventricular haemorrhage

eResults – Secondary Outcomes

1. Bronchopulmonary Dysplasia
Network meta-analysis was performed using a fixed-effects model as it was more conservative. DIC for the fixed-effect model was 91.45, random-effect model 92.9. Model used for the direct comparisons along with odds ratio for each comparison, both network and direct are shown in eTable 3a.

eTable 5. Odds Ratio for Both the Direct and Network Comparisons For Secondary Outcomes

Bronchopulmonary Dysplasia	Prophylaxis	Threshold 30%	Threshold 40%	Threshold 50%	Threshold 60%
**Prophylaxis**		1.4[0.87,2.23] &	0.77[0.37,1.61] ^	0.93[0.74,1.16] *	1.02[0.71,1.45] *
**Threshold 30%**	1.39[0.87,2.24]		-	-	-
**Threshold 40%**	0.77[0.37,1.58]	0.55[0.23,1.30]		-	-
**Threshold 50%**	0.93[0.74,1.16]	0.66[0.39,1.12]	1.20[0.57,2.61]		-
**Threshold 60%**	1.02[0.72,1.45]	0.73[0.40,1.32]	1.32[0.60,3.01]	1.10[0.72,1.67]	

eTable 5a. Odds ratio for both the direct and network comparison for the outcome bronchopulmonary dysplasia.
Network meta-analysis comparisons are below the greyed line, direct comparisons are above the line.
Comparisons that did not reach statistical significance (credibility intervals for the odds ratio cross 1) are highlighted in yellow. Comparisons that do reach statistical significance are highlighted in blue.
Most conservative method of analysis was used in each case.
*denotes fixed effect model, # denotes random effect model for direct comparisons, & denotes only one study in comparison with no convergence of random effect model – fixed effect used, ^ denotes zero events in one arm of one study leading to use of the fixed effect model

2. Chronic Lung Disease
Network meta-analysis was performed using a random-effects model, as the most conservative model.
DIC for the fixed-effect model was 109, random-effect model 110.7. Median between-study standard deviation 0.1751 (95% CrI 0.0078, 0.8729), variance 0.031.
Models used in the direct comparisons with odds ratio for each comparison are shown in table 3.

Chronic Lung Disease	Prophylaxis	Threshold 30%	Threshold 40%	Threshold 50%	Threshold 60%
**Prophylaxis**		1.47[0.07,30.69] #	1.07[0.8,1.43] *	3.97[0.88,30.78] &	0.6[0.33,1.06] *
**Threshold 30%**	1.48[0.82,2.63]		-	-	-
**Threshold 40%**	1.05[0.63,1.64]	0.71[0.32,1.48]		-	-

**Threshold 50%**	4.08[0.77,35.45]	2.75[0.46,25.87]	3.90[0.69,35.98]		-
**Threshold 60%**	0.59[0.28,1.22]	0.40[0.16,1.01]	0.56[0.23,1.36]	0.14[0.01,0.91]	

eTable 5b. Odds ratio for both the direct and network comparisons for CLD. Description of table as per table 3a

3. Bronchopulmonary Dysplasia or Chronic Lung Disease at maximal follow up
For this outcome, a random-effect model was used for the network meta-analysis as the more conservative choice.
DIC for the fixed model was 152.9, random model 154.7. Median between study deviation 0.1619 (95% CrI 0.0071, 0.678), variance 0.26.
Models used in the direct comparisons along with odds ratio for each comparison are shown in eTable 3c.

CLD or BPD	Prophylaxis	Threshold 30%	Threshold 40%	Threshold 50%	Threshold 60%
**Prophylaxis**		1.45[1.09,1.93] *	0.94[0.68,1.29] ^	0.93[0.74,1.16] ^	0.91[0.65,1.26] *
**Threshold 30%**	1.45[0.95,2.21]		-	-	-
**Threshold 40%**	0.91[0.54,1.41]	0.63[0.32,1.13]		-	-
**Threshold 50%**	0.96[0.59,2.00]	0.66[0.36,1.58]	1.06[0.57,2.75]		-
**Threshold 60%**	0.86[0.47,1.34]	0.59[0.28,1.06]	0.94[0.46,1.81]	0.90[0.32,1.64]	

eTable 5c. Odds ratio for the comparisons of both the direct and network comparisons for CLD or BPD. Description of table as per eTable 3a

4. Pneumothorax (or other air-leak)
Network meta-analysis was performed using a random-effects model, as the most conservative model.
DIC for the fixed-effect model was 159.5, random-effect model was 154.3. Between study standard deviation was 0.859 (95% CrI 0.197, 2.115), variance 0.74.
Models used in the direct comparisons along with odds ratio for each comparison are shown in eTable 3d.

Pneumothorax	Prophylaxis	Threshold 30%	Threshold 40%	Threshold 50%	Threshold 60%
**Prophylaxis**		2.36[0.29,22.15] #	1.38[0.89,2.17] *	0.92[0.63,1.35] *	1.67[0.88,3.19] *
**Threshold 30%**	2.41[0.61,10.48]		-	-	-
**Threshold 40%**	1.26[0.42,3.97]	0.52[0.08,3.13]		-	-
**Threshold 50%**	0.81[0.19,3.47]	0.33[0.04,2.49]	0.64[0.10,3.99]		-
**Threshold 60%**	2.05[0.50,10.72]	0.85[0.11,7.42]	1.62[0.27,12.07]	2.54[0.35,23.13]	

eTable 5d. Odds ratio for the comparisons of both the direct and network comparisons for pneumothorax. Description of table as per eTable 3a

5. Surfactant Treatment (proportion requiring surfactant)
Network meta-analysis not performed.
Proportions receiving surfactant (binary): 99.07% of the prophylaxis group received any surfactant
41.54% in the 30% group
53.82% in the 40% group
64.42% in the 50% group
46.22% of 60% group.

6. Number of Surfactant Doses Required
Network meta-analysis was performed using a random-effects model, as the most conservative model.
DIC for the fixed-effect model was 270, random-effect model 269. Between study standard deviation 2.504 (95% CrI 0.1212, 4.879), variance 6.27.
Models used in the direct comparisons along with odds ratio for each comparison are shown in eTable 3f.

Surfactant - Number of Doses	Prophylaxis	Threshold 30%	Threshold 40%	Threshold 50%	Threshold 60%
Prophylaxis		0.51[0.46,0.56] *	0.71[0.63,0.8] *	0.65[0.58,0.73] *	0.26[0.21,0.32] *
Threshold 30%	0.51[0.46,0.56]		-	-	-
Threshold 40%	0.71[0.63,0.81]	1.41[1.20,1.65]		-	-
Threshold 50%	0.65[0.58,0.73]	1.29[1.10,1.51]	0.91[0.77,1.08]		-
Threshold 60%	0.26[0.21,0.32]	0.52[0.41,0.65]	0.37[0.29,0.47]	0.40[0.32,0.51]	

eTable 5e. Odds ratio for the comparisons of both the direct and network comparisons for number of surfactant doses required. Description of table as per eTable 3a

7. Total Number of Major Morbidities
Network meta-analysis was performed using a fixed-effects model, as the most conservative model. DIC for the fixed-effect model was 168.5, random-effect model 168.5.
Models used in the direct comparisons along with odds ratio for each comparison are shown in eTable 3g.

Major Morbidity	Prophylaxis	Threshold 30%	Threshold 40%	Threshold 50%	Threshold 60%
Prophylaxis		1.14[0.93,1.4] *	1.18[0.89,1.56] *	1.04[0.92,1.18] *	1.02[0.81,1.28] *
Threshold 30%	1.14[0.94,1.40]		-	-	-
Threshold 40%	1.18[0.89,1.56]	1.03[0.73,1.45]		-	-
Threshold 50%	1.04[0.92,1.18]	0.91[0.72,1.16]	0.89[0.65,1.20]		-

**Threshold 60%**	1.02[0.81,1.28]	0.89[0.65,1.21]	0.86[0.60,1.24]	0.97[0.75,1.27]	

eTable 5f. Odds ratio for the comparisons of both the direct and network comparisons for total number of major morbidities. Description of table as per eTable 3a

8. Grade 3 or 4 Intraventricular Haemorrhage

Network meta-analysis was performed using a random-effects model, as the most conservative model.

DIC for the fixed-effect model was 138.3, random-effect model 137.8. Between study standard deviation 0.449 (95% CrI 0.326, 1.281), variance 0.2.

Models used in the direct comparisons along with odds ratio for each comparison are shown in eTable 3h.

Grade 3 or 4 Intraventricular Haemorrhage	Prophylaxis	Threshold 0.3	Threshold 0.4	Threshold 0.5	Threshold 0.6
**Prophylaxis**		2.16[0.14,34.19] #	1.59[0.91,2.84] *	1.21[0.9,1.63] &	0.67[0.32,1.32] &
**Threshold 0.3**	2.01[0.83,5.46]		-	-	-
**Threshold 0.4**	1.69[0.77,4.10]	0.84[0.24,2.93]		-	-
**Threshold 0.5**	1.11[0.44,2.47]	0.55[0.14,1.75]	0.65[0.18,1.94]		-
**Threshold 0.6**	0.68[0.22,2.03]	0.34[0.07,1.35]	0.40[0.09,1.52]	0.61[0.16,2.60]	

eTable 5g. Odds ratio for the comparisons of both the direct and network comparisons for grade 3 or 4 intraventricular haemorrhage. Description of table as per eTable 3a

9. Periventricular Leukomalacia

Network meta-analysis was performed using a fixed-effect model, as the most conservative model. DIC for the fixed-effect model was 78.82, random-effect model 80.17.

Models used in the direct comparisons along with odds ratio for each comparison are shown in eTable 3i.

Periventricular Leucomalacia	Prophylaxis	Threshold 30%	Threshold 40%	Threshold 50%	Threshold 60%
**Prophylaxis**		0.81[0.51,1.28] *	0.63[0.07,4.18] *	0.81[0.22,2.77] *	0.58[0.2,1.5] *
**Threshold 30%**	0.81[0.51,1.28]		-	-	-
**Threshold 40%**	0.64[0.07,4.25]	0.79[0.09,5.53]		-	-
**Threshold 50%**	0.80[0.21,2.81]	0.98[0.25,3.79]	1.26[0.13,14.92]		-
**Threshold 60%**	0.58[0.19,1.50]	0.71[0.22,2.06]	0.91[0.10,9.56]	0.72[0.14,3.64]	

eTable 5h. Odds ratio for the comparisons of both the direct and network comparisons for periventricular leukomalacia. Description of table as per eTable 3a

10. Necrotising Enterocolitis

Network meta-analysis was performed using a fixed-effects model, as the most conservative model. DIC for the fixed-effect model was 112.5, random-effect model 114.4.
Models used in the direct comparisons along with odds ratio for each comparison are shown in eTable 3j.

Necrotising Enterocolitis	Prophylaxis	Threshold 30%	Threshold 40%	Threshold 50%	Threshold 60%
**Prophylaxis**		0.86[0.54,1.36] *	1.27[0.81,2] ^	1.27[0.92,1.77] *	1.15[0.61,2.08] &
**Threshold 30%**	0.86[0.55,1.35]		-	-	-
**Threshold 40%**	1.27[0.81,2.01]	1.48[0.78,2.80]		-	-
**Threshold 50%**	1.27[0.91,1.77]	1.48[0.84,2.59]	1.00[0.57,1.74]		-
**Threshold 60%**	1.15[0.61,2.10]	1.33[0.61,2.84]	0.90[0.41,1.91]	0.90[0.44,1.80]	

eTable 5i. Odds ratio for the comparisons of both the direct and network comparisons for necrotising enterocolitis. Description of table as per eTable 3a

11. Retinopathy of Prematurity

Network meta-analysis was performed using a random-effects model, as the most conservative model.
DIC for the fixed-effect model was 65.68, random-effect model 67.53. Between study standard deviation 0.517 (95% CrI 0.0198, 3.845), variance 0.27.
Models used in the direct comparisons along with odds ratio for each comparison are shown in eTable 3k.

Retinopathy of Prematurity > Stage 2	Prophylaxis	Threshold 30%	Threshold 40%	Threshold 50%	Threshold 60%
**Prophylaxis**		1.02[0.03,37.98] &	0.9[0.34,2.31] *	1.01[0.72,1.41] *	2.35[1.02,5.42] &
**Threshold 30%**	1.01[0.01,96.83]		-	-	-
**Threshold 40%**	0.87[0.09,7.05]	0.85[0.01,117.92]		-	-
**Threshold 50%**	0.99[0.12,6.96]	0.97[0.01,121.39]	1.14[0.06,23.17]		-
**Threshold 60%**	2.36[0.13,40.29]	2.31[0.01,464.98]	2.69[0.07,101.80]	2.38[0.07,76.63]	

eTable 5j. Odds ratio for the comparisons of both the direct and network comparisons for retinopathy of prematurity greater than stage 2. Description of table as per eTable 3a

BMJ Publishing Group Limited (BMJ) disclaims all liability and responsibility arising from any reliance placed on this supplemental material which has been supplied by the author(s)

References

1. Mills EJ, Ioannidis JP, Thorlund K, Schünemann HJ, Puhan MA, Guyatt GH (2012) How to use an article reporting a multiple treatment comparison meta-analysis. Jama 308 (12):1246-1253. doi:10.1001/2012.jama.11228
2. Dias S, Sutton AJ, Ades AE, Welton NJ (2013) Evidence synthesis for decision making 2: a generalized linear modeling framework for pairwise and network meta-analysis of randomized controlled trials. Med Decis Making 33 (5):607-617. doi:10.1177/0272989x12458724
3. Chaimani A, Higgins JP, Mavridis D, Spyridonos P, Salanti G (2013) Graphical tools for network meta-analysis in STATA. PLoS One 8 (10):e76654. doi:10.1371/journal.pone.0076654
4. Salanti G, Ades AE, Ioannidis JP (2011) Graphical methods and numerical summaries for presenting results from multiple-treatment meta-analysis: an overview and tutorial. J Clin Epidemiol 64 (2):163-171. doi:10.1016/j.jclinepi.2010.03.016
5. Dias S, Welton N, Sutton A, Ades A (2016) NICE DSU technical support document 2: a generalised linear modelling framework for pairwise and network meta-analysis of randomised controlled trials (Update Sept 2016). NICE DSU
6. Alallah J (2012) Early CPAP versus surfactant in extremely preterm infants. Journal of Clinical Neonatology 1 (1):12-13. doi:http://dx.doi.org/10.4103/2249-4847.92233
7. Bahadue FL, Soll R (2012) Early versus delayed selective surfactant treatment for neonatal respiratory distress syndrome. Cochrane Database of Systematic Reviews (11). doi:10.1002/14651858.CD001456.pub2
8. Bancalari E (2012) Non-invasive neonatal respiratory support. Journal of Maternal-Fetal and Neonatal Medicine 25:2. doi:http://dx.doi.org/10.3109/14767058.2012.679162
9. Corbet A (1993) CLINICAL-TRIALS OF SYNTHETIC SURFACTANT IN THE RESPIRATORY-DISTRESS SYNDROME OF PREMATURE-INFANTS. Clin Perinatol 20 (4):737-760
10. Dani C (2016) Surfactant Treatment Threshold during NCPAP for the Treatment of Preterm Infants with Respiratory Distress Syndrome. Am J Perinatol 33 (10):925-929. doi:http://dx.doi.org/10.1055/s-0036-1582395
11. Carnielli VP, Ancona OR (2020) Medium vs Low Oxygen Threshold for the Surfactant Administration. https://ClinicalTrials.gov/show/NCT04199364,
12. Iarukova N, Chernev T, Nikolov A (1998) [The use of Curosurf with premature infants--the prevention or treatment of the neonatal RDS]. Akush Ginekol (Sofiia) 37 (3):12-14
13. Mostovoĭ AV, Aleksandrovich I, Sapun OI, Trifonova EG, Tret'iakova EP, Bogdanova RZ, Karpova AL (2009) Effect of surfactant administration time on the outcomes in low and extremely low birth weight neonates. Anesteziologiia i reanimatologiia (1):43-46
14. Alba J, Agarwal R, Hegyi T, Hiatt IM (1995) EFFICACY OF SURFACTANT THERAPY IN INFANTS MANAGED WITH CPAP. Pediatric Pulmonology 20 (3):172-176. doi:10.1002/ppul.1950200308
15. Billman D, Nicks J, Schumacher R (1994) Exosurf rescue surfactant improves high ventilation-perfusion mismatch in respiratory distress syndrome. Pediatric pulmonology 18 (5):279-283
16. Bower LK, Barnhart SL, Betit P, Hendon B, MasiLynch J, Wilson BG (1996) Surfactant replacement therapy (Reprinted from Respiratory Care, vol 39, pg 824-829, 1994). Int Anesthesiol Clin 34 (1):153-161. doi:10.1097/00004311-199603410-00019
17. Bunt JE, Carnielli VP, Janssen DJ, Wattimena JL, Hop WC, Sauer PJ, Zimmermann LJ (2000) Treatment with exogenous surfactant stimulates endogenous surfactant synthesis in premature infants with respiratory distress syndrome. Critical care medicine 28 (10):3383-3388. doi:10.1097/00003246-200010000-00001
18. Dunn MS, Shennan AT, Hoskins EM, Lennox K, Enhorning G (1988) Two-year follow-up of infants enrolled in a randomized trial of surfactant replacement therapy for prevention of neonatal respiratory distress syndrome. Pediatrics 82 (4):543-547
19. Egberts J, De Winter JP, Van Sonderen L, Van Den Anker JN (1994) Theoretical calculation of the cost for neonatal care after any prophylaxis or theraperutical administration of surfactant. [Dutch] (Een theoretische berekening van de kosten van de neonatale zorg na een eventueel profylactisch of therapeutisch gebruik van surfactant.). Tijdschrift voor Kindergeneeskunde 62 (2):97-103
20. Gore SM (1993) OSIRIS trial. Lancet (london, england) 341 (8838):172; author reply 173-174
21. Hanssler L, Zhou C, Roll C, Wiesemann HG (1994) Effects of exogenous surfactant therapy on lung function in mechanically ventilated preterm infants with severe respiratory distress syndrome (RDS). [German] (Effekte der surfactant-substitution auf die lungenfunktion beatmeter fruhgeborener mit schwerem atemnotsyndrom.). Pediatrics and Related Topics 32 (1):31-39

22. Hentschel R, Dittrich F, Hilgendorff A, Wauer R, Westmeier M, Gortner L (2009) Neurodevelopmental outcome and pulmonary morbidity two years after early versus late surfactant treatment: does it really differ? Acta Paediatr 98 (4):654-659. doi:10.1111/j.1651-2227.2008.01216.x
23. Keller RL, Eichenwald EC, Hibbs AM, Rogers EE, Wai KC, Black DM, Ballard PL, Asselin JM, Truog WE, Merrill JD, Mammel MC, Steinhorn RH, Ryan RM, Durand DJ, Bendel CM, Bendel-Stenzel EM, Courtney SE, Dhanireddy R, Hudak ML, Koch FR, Mayock DE, McKay VJ, Helderman J, Porta NF, Wadhawan R, Palermo L, Ballard RA, Grp TS (2017) The Randomized, Controlled Trial of Late Surfactant: Effects on Respiratory Outcomes at 1-Year Corrected Age. Journal of Pediatrics 183:19-+. doi:10.1016/j.jpeds.2016.12.059
24. Keller RL, Rogers E, Eichenwald E, Hibbs A, Black D, Ballard P, Ballard R (2016) One year pulmonary outcomes in the trial of late surfactant (TOLSURF). Journal of Investigative Medicine 64 (1):250-251. doi:http://dx.doi.org/10.1136/jim-d-15-00013.268
25. Kim SM, Park YJ, Chung SH, Choi YS, Kim CH, Bae CW (2014) Early prophylactic versus late selective use of surfactant for respiratory distress syndrome in very preterm infants: a collaborative study of 53 multi-center trials in Korea. J Korean Med Sci 29 (8):1126-1131. doi:10.3346/jkms.2014.29.8.1126
26. Kong X, Cui Q, Hu Y, Huang W, Ju R, Li W, Wang R, Xia S, Yu J, Zhu T, Feng Z (2016) Bovine Surfactant Replacement Therapy in Neonates of Less than 32 Weeks' Gestation: A Multicenter Controlled Trial of Prophylaxis versus Early Treatment in China--a Pilot Study. Pediatr Neonatol 57 (1):19-26. doi:10.1016/j.pedneo.2015.03.007
27. Lefort S, Diniz EMA, Vaz FAC (1999) A follow-up clinical trial involving preterm neonates at risk for Respiratory Distress Syndrome (RDS), submitted to prophylactic surfactant of porcine origin comparing two different dosage regimens. 4th World Congress of Perinatal Medicine. Medimond S R L, 40128 Bologna
28. Morley C (1993) OSIRIS trial. Lancet 341 (8838):172-173; author reply 173-174
29. Robertson B (1990) European multicenter trials of curosurf for treatment of neonatal respiratory distress syndrome. Lung 168 Suppl:860-863. doi:10.1007/bf02718220
30. Robertson B, Curstedt T, Tubman R, Strayer D, Berggren P, Kok J, Koppe J, van Sonderen L, Halliday H, McClure G, et al. (1992) A 2-year follow up of babies enrolled in a European multicentre trial of porcine surfactant replacement for severe neonatal respiratory distress syndrome. Collaborative European Multicentre Study Group. Eur J Pediatr 151 (5):372-376. doi:10.1007/bf02113261
31. Robertson B, Speer CP (1993) OSIRIS trial. Lancet 341 (8838):172; author reply 173-174
32. Sanghvi KP, Merchant RH (1998) Single dose surfactant rescue therapy in neonatal respiratory distress syndrome. Indian Pediatr 35 (6):533-536
33. Svenningsen NW, Saugstad OD (1996) Surfactant treatment of extremely low birth weight (ELBW) infants. Acp 6 (1):11-17
34. Tarnow-Mordi W (1993) OSIRIS trial. Lancet 341 (8838):174
35. Wagner CL, Kramer BM, Kendig JW, Brooks JG, Cox C, Wagner MT, Phelps DL (1995) SCHOOL-AGE FOLLOW-UP OF A SINGLE-DOSE PROPHYLACTIC SURFACTANT COHORT. J Dev Behav Pediatr 16 (5):327-332
36. Ware J, Taeusch HW, Soll RF, McCormick MC (1990) Health and developmental outcomes of a surfactant controlled trial: follow-up at 2 years. Pediatrics 85 (6):1103-1107
37. Anonymous (1992) Early versus delayed neonatal administration of a synthetic surfactant - The judgment of OSIRIS. Lancet 340 (8832):1363-1369. doi:http://dx.doi.org/10.1016/0140-6736%2892%2992557-V
38. Anonymous (1992) Early or selective surfactant (colfosceril palmitate, Exosurf) for intubated babies at 26 to 29 weeks gestation. A European double-blind trial with sequential analysis. European Exosurf Study Group. The Online journal of current clinical trials Doc No 28:[3886 words; 3847 paragraphs]
39. Chu GL, Wang J, Xin Y, Zheng J, Zheng RX, Bi DZ (2006) Protective and curative effects of prophylactic administration of pulmonary surfactant on neonatal respiratory distress syndrome. [Chinese]. National Medical Journal of China 86 (13):876-880
40. Dunn MS, Shennan AT, Zayack D, Possmayer F (1991) Bovine surfactant replacement therapy in neonates of less than 30 weeks' gestation: A randomized controlled trial of prophylaxis versus treatment. Pediatrics 87 (3):377-386
41. Rong ZH, Chang LW, Cheng HB, Wang HZ, Zhu XF, Peng F, Fan QH, Lu W, Pan R, Xiong L, Jiao R, Sun J, Xia SW, Xie JJ (2019) A Multicentered Randomized Study on Early versus Rescue Calsurf Administration for the Treatment of Respiratory Distress Syndrome in Preterm Infants. Am J Perinatol 36 (14):1492-1497. doi:10.1055/s-0039-1678530

42. Verder H, Robertson B, Greisen G, Ebbesen F, Albertsen P, Lundstrom K, Jacobsen T (1994) Surfactant therapy and nasal continuous positive airway pressure for newborns with respiratory distress syndrome. Danish-Swedish Multicenter Study Group. N Engl J Med 331 (16):1051-1055. doi:10.1056/nejm199410203311603
43. Anonymous (2004) Early surfactant for neonates with mild to moderate respiratory distress syndrome: A multicenter, randomized trial. Journal of Pediatrics 144 (6):804-808. doi:http://dx.doi.org/10.1016/j.jpeds.2004.03.024
44. Bevilacqua G, Halliday H, Parmigiani S, Robertson B (1993) Randomized multicentre trial of treatment with porcine natural surfactant for moderately severe neonatal respiratory distress syndrome. Journal of Perinatal Medicine 21 (5):329-340
45. Bevilacqua G, Parmigiani S, Robertson B (1996) Prophylaxis of respiratory distress syndrome by treatment with modified porcine surfactant at birth: A multicentre prospective randomized trial. Journal of Perinatal Medicine 24 (6):609-620. doi:10.1515/jpme.1996.24.6.609
46. Centre Hospitalier Universitaire A (2011) Surfactant Versus Nasal Continuous Positive Airway Pressure (nCPAP) for Respiratory Distress Syndrome in the Newborn ≥ 35 Weeks of Gestation. https://ClinicalTrials.gov/show/NCT01306240,
47. Chen JY (1990) Exogenous surfactant for treatment of respiratory distress syndrome in premature infants. Journal of the Formosan Medical Association / Taiwan yi zhi 89 (2):110-114
48. Chile PUCd (2007) Early CPAP in Respiratory Distress Syndrome. https://ClinicalTrials.gov/show/NCT00368680,
49. Chile PUCd, Pediatría SCd (2006) Two Strategies of RDS Treatment in Newborns With Birth Weight > 1500 Grams. https://ClinicalTrials.gov/show/NCT00277030,
50. China Medical University C, Hospital NTU, Hospital TMU, Hospital CG, Hospital CGM (2009) Prevention of Chronic Lung Disease (CLD) in Preterm Infants. https://ClinicalTrials.gov/show/NCT00883532,
51. Cologne Uo, Schleswig-Holstein Uo, Hospital ACs, Bochum RUo, Datteln VK-uJ, Leverkusen Ho, Kliniken der Stadt Koeln KR, Heinrich-Heine University D, Aschaffenburg-Alzenau K, GmbH AKH, Stuttgart K, Siegen DK, University Hospital B, Charite University B, Germany, Cologne TCTC, Education GFMo, Research (2009) Surfactant Application During Spontaneous Breathing With Continuous Positive Airway Pressure (CPAP) in Premature Infants < 27 Weeks. https://ClinicalTrials.gov/show/NCT00751959,
52. CTRI/2008/091/000234 (2009) Early Surfactant Therapy to prevent need for ventilation for preterm infants(gestation <34weeks)on Bubble CPAP. (trans: No sponsor N).
53. CTRI/2015/07/005968 (2015) Exogenous surfactant obtained from goat lungs for treating respiratory difficulty due to lack of surfactant in preterm neonates (trans: All India Institute of Medical Sciences New Delhi Y).
54. Escobedo MB, Gunkel JH, Kennedy KA, Shattuck KE, Sanchez PJ, Seidner S, Hensley G, Cochran CK, Moya F, Morris B, Denson S, Stribley R, Naqvi M, Lasky RE, Texas Neonatal Res G (2004) Early surfactant for neonates with mild to moderate respiratory distress syndrome: A multicenter, randomized trial. Journal of Pediatrics 144 (6):804-808. doi:10.1016/j.jpeds.2004.03.024
55. Finer NN, Carlo WA, Duara S, Fanaroff AA, Donovan EF, Wright LL, Kandefer S, Poole WK, Natl Inst Child Hlth Human Dev N (2004) Delivery room continuous positive airway pressure/positive end-expiratory pressure in extremely low birth weight infants: A feasibility trial. Pediatrics 114 (3):651-657. doi:10.1542/peds.2004-0394
56. Gopel W, Kribs A, Laux R, Hohn T, Wieg C, Kattner E, Avenarius S, Von der Wense A, Vochem M, Groneck P, Weller U, Moller J, Roth B, Herting E (2010) SURFACTANT TREATMENT OF SPONTANEOUSLY BREATHING PRETERM INFANTS TO AVOID MECHANICAL VENTILATION - A RANDOMIZED CONTROLLED TRIAL. Pediatric Research 68:21-21
57. Gortner L, Bernsau U, Hellwege HH, Hieronimi G, Jorch G, Reiter HL (1990) A multicenter randomized controlled clinical trial of bovine surfactant for prevention of respiratory distress syndrome. Lung 168 (SUPPL.):864-869
58. Gortner L, Wauer RR, Hammer H, Stock GJ, Heitmann F, Reiter HL, Kuhl PG, Moller JC, Friedrich HJ, Reiss I, Hentschel R, Jorch G, Hieronimi G, Kuhls E (1998) Early versus late surfactant treatment in preterm infants of 27 to 32 weeks' gestational age: a multicenter controlled clinical trial. Pediatrics 102 (5):1153-1160. doi:10.1542/peds.102.5.1153
59. Goto A (1988) Surfactant replacement therapy in neonatal RDS. Multicentre, randomized controlled trial. Yokohama Medical Bulletin 39 (5-6):211-217
60. Hascoet JM, ARAIRLOR, S.p.A. CF, Universitaire MR (2009) Exogenous Surfactant in Very Preterm Neonates in Prevention of Bronchopulmonary Dysplasia. https://ClinicalTrials.gov/show/NCT01039285,

61. Horn AR, Pieper C, Els I, Holgate S (2009) Early surfactant therapy and nasal continuous positive airways pressure for mild respiratory distress syndrome - A pilot study. SAJCH South African Journal of Child Health 3 (2):48-54
62. Hospital D, University tRIoSotTMM (2016) Surfactant Administration in Preterm Infants. https://ClinicalTrials.gov/show/NCT02821273,
63. Hospital D, University tRIoSotTMM (2017) Surfactant for Neonate With Acute Respiratory Distress Syndrome (ARDS). https://ClinicalTrials.gov/show/NCT03217162,
64. Institute OHR (2013) A Multi-center Trial to Determine if Curosurf® Reduces the Duration of Mechanical Ventilation in Very Preterm Infants. https://ClinicalTrials.gov/show/NCT01709409,
65. IRCT138905104486N1 (2010) Effect of prophylactic surfactant on outcome of premature neonate (trans: Kermanshah University of Medical Sciences N).
66. IRCT201205213512N2 (2012) The effect of the use of surfactant administration during NCPAP treatment on complications of RDS (trans: Mazandaran University of Medical Siences N).
67. IRCT20120728010430N8 (2019) Investigating CPAP in Treatment of RDS (trans: Esfahan University of Medical Sciences N).
68. Kattwinkel J, Bloom BT, Delmore P, Glick C, Brown D, Lopez S, Willett L, Egan EA, Conaway M, Patrie J (2000) High-versus low-threshold surfactant retreatment for neonatal respiratory distress syndrome. Pediatrics 106 (2 Pt 1):282-288. doi:10.1542/peds.106.2.282
69. Keller RL, Merrill JD, Black DM, Steinhorn RH, Eichenwalds EC, Durand DJ, Ryan RM, Truog WE, Courtney SE, Ballard PL, Ballard RA (2012) Late administration of surfactant replacement therapy increases surfactant protein-B content: a randomized pilot study. Pediatric Research 72 (6):613-619. doi:10.1038/pr.2012.136
70. Kendig JW, Ryan RM, Sinkin RA, Maniscalco WM, Notter RH, Guillet R, Cox C, Dweck HS, Horgan MJ, Reubens LJ, et al. (1998) Comparison of two strategies for surfactant prophylaxis in very premature infants: a multicenter randomized trial. Pediatrics 101 (6):1006-1012. doi:10.1542/peds.101.6.1006
71. Konishi M, Fujiwara T, Chida S, Maeta H, Shimada S, Kasai T, Fuji Y, Murakami Y (1992) A prospective, randomized trial of early versus late administration of a single dose of surfactant-TA. Early Human Development 29 (1-3):275-282. doi:http://dx.doi.org/10.1016/0378-3782%2892%2990164-C
72. Mahmoud I, Raheleh D, Manijeh K, Azizollah A (2013) Comparison of Nasal Continuous Positive Airway Pressure Therapy with and without Prophylactic Surfactant in Preterm Neonates. Iranian Journal of Neonatology 4 (3):26-34
73. Manitoba Uo (2012) Early CPAP And Large Volume Minimally Invasive Surfactant (ECALMIST) in Preterm Infants With RDS. https://ClinicalTrials.gov/show/NCT01553292,
74. Manitoba Uo (2013) ECALMIST Versus InSurE in Preterm Infant < 32 Weeks,Multicenter, Multinational RCT. https://ClinicalTrials.gov/show/NCT01848262,
75. Morley CJ, Davis PG, Doyle LW, Brion LP, Hascoet JM, Carlin JB (2008) Nasal CPAP or intubation at birth for very preterm infants. New England Journal of Medicine 358 (7):700-708. doi:http://dx.doi.org/10.1056/NEJMoa072788
76. Nakhshab M, Tajbakhsh M, Khani S, Farhadi R (2015) Comparison of the effect of surfactant administration during nasal continuous positive airway pressure with that of nasal continuous positive airway pressure alone on complications of respiratory distress syndrome: a randomized controlled study. Pediatr Neonatol 56 (2):88-94. doi:10.1016/j.pedneo.2014.05.006
77. Nct (2005) SURFAXIN® Treatment for Prevention of Bronchopulmonary Dysplasia (BPD) in Very Low Birth Weight (VLBW) Infants. https://clinicaltrialsgov/show/NCT00215540
78. Nct (2007) Curosurf and Survanta Treatment(CAST)of RDS in Very Premature Infants. https://clinicaltrialsgov/show/NCT00767039
79. NCT03217162 (2017) Surfactant for Neonate With Acute Respiratory Distress Syndrome (ARDS) (trans: Daping H, the Research Institute of Surgery of the Third Military Medical University Y).
80. Okulu E, Arsan S, Akin IM, Atasay B, Alan S, Kilic A, Turmen T (2011) COMPARISON OF TWO STRATEGIES FOR SURFACTANT PROPHYLAXIS IN PREMATURE INFANTS: PRELIMINARY DATA OF A RANDOMIZED TRIAL. Pediatric Research 70:535-535. doi:10.1038/pr.2011.760
81. Okulu E, Arsan S, Akin IM, Kilic A, Alan S, Atasay B (2012) The timing of surfactant prophylaxis in very-low-birth-weight preterms: Is earlier better? Early Human Development 88:S110
82. Okulu E, Arsan S, Akin IM, Kilic A, Alan S, Atasay B (2012) The timing of surfactant prophylaxis in very-lowbirth-weight preterms: Is earlier better? Archives of Disease in Childhood 97:A67. doi:http://dx.doi.org/10.1136/archdischild-2012-302724.0232

83. Okulu E, Arsan S, Mungan Akin I, Alan S, Kilic A, Atasay B (2015) Early or later prophylactic INSURE in preterm infants of less than 30 weeks' gestation. Turk J Pediatr 57 (1):1-8
84. Plavka R, Kopecky P, Sebron V, Leiska A, Svihovec P, Ruffer J, Dokoupilova M, Zlatohlavkova B, Janus V, Keszler M (2002) Early versus delayed surfactant administration in extremely premature neonates with respiratory distress syndrome ventilated by high-frequency oscillatory ventilation. Intensive Care Med 28 (10):1483-1490. doi:10.1007/s00134-002-1440-1
85. Research ZTBWsH, Hospital E (2010) Surfactant Administration During Spontaneous Breathing. https://ClinicalTrials.gov/show/NCT01329432,
86. Stevenson D, Walther F, Long W, Sell M, Pauly T, Gong A, Easa D, Pramanik A, LeBlanc M, Anday E, et al. (1992) Controlled trial of a single dose of synthetic surfactant at birth in premature infants weighing 500 to 699 grams. The American Exosurf Neonatal Study Group I. J Pediatr 120 (2 Pt 2):S3-12. doi:10.1016/s0022-3476(05)81226-0
10.1016/s0022-3476(05)81226-0.
87. Tapia JL, Urzua S, Bancalari A, Meritano J, Torres G, Fabres J, Toro CA, Rivera F, Cespedes E, Burgos JF, Mariani G, Roldan L, Silvera F, Gonzalez A, Dominguez A (2012) Randomized Trial of Early Bubble Continuous Positive Airway Pressure for Very Low Birth Weight Infants. The Journal of Pediatrics 161 (1):75-80.e71. doi:https://doi.org/10.1016/j.jpeds.2011.12.054
88. Therapeutics W (2005) SURFAXIN® Treatment for Prevention of Bronchopulmonary Dysplasia (BPD) in Very Low Birth Weight (VLBW) Infants. https://ClinicalTrials.gov/show/NCT00215540,
89. Uk C, Trust LTHNHS, London IC (2018) The Effect of Surfactant Dose on Outcomes in Preterm Infants With RDS. https://ClinicalTrials.gov/show/NCT03808402,
90. University A (2010) Comparison of Two Strategies for Surfactant Prophylaxis in Premature Infants. https://ClinicalTrials.gov/show/NCT01294852,
91. University of California SF, National Heart L, Institute B (2010) Trial of Late Surfactant for Prevention of Bronchopulmonary Dysplasia. https://ClinicalTrials.gov/show/NCT01022580,
92. University XHoCS (2007) Early NCPAP Before Surfactant Treatment in Very Preterm Infants With RDS. https://ClinicalTrials.gov/show/NCT01996670,
93. Verder H, Albertsen P, Ebbesen F, Greisen G, Robertson B, Bertelsen A, Agertoft L, Djernes B, Nathan E, Reinholdt J (1999) Nasal continuous positive airway pressure and early surfactant therapy for respiratory distress syndrome in newborns of less than 30 weeks' gestation. Pediatrics 103 (2):E24. doi:10.1542/peds.103.2.e24
94. Verder H, Ebbesen F, Fenger-Gron J, Henriksen TB, Andreasson B, Bender L, Bertelsen A, Bjorklund LJ, Dahl M, Esberg G, Eschen C, Hovring M, Kreft A, Kroner J, Lundberg F, Pedersen P, Reinholdt J, Stanchev H (2013) Early surfactant guided by lamellar body counts on gastric aspirate in very preterm infants. Neonatology 104 (2):116-122. doi:10.1159/000351638
10.1159/000351638. Epub 2013 Jul 9.
95. Wauer RR, Schmalisch G, Abel, Arandt, Eckert, Hock S, Jahrig D, Klaube, Meier, Plath, Rustow, Schmalisch, Schwerecke, Thummler, Tiller, Topke B, Vogtmann, Wauer (1996) Retrospective analysis of a preterm stopped controlled randomized multicentre rescue trial of neonatal respiratory distress syndrome using bovine surfactant. [German] (Retrospektive analyse einer vorzeitig abgebrochenen klinischen kontrollierten studie zur therapie des neonatalen atemnotsyndroms mit einem bovinen surfactant-praparat.). Pediatrics and Related Topics 34 (5):337-352
96. Yekta M, Research ZTBWsH, Hospital E (2012) Comparison of Effectiveness of Nasal CPAP and Nasal IMV in Early Rescue Surfactant Treatment in Preterm Infants. https://ClinicalTrials.gov/show/NCT01741129,
97. Ctri (2009) Early Surfactant Therapy to prevent need for ventilation for preterm infants(gestation <34weeks)on Bubble CPAP (trans: No sponsor N).
98. Finer NN (2011) SUPPORT trial: Focussing on ROP and BPD. Early CPAP vs. surfactant in extremely preterm infants. Monatsschrift fur Kinderheilkunde 159:22. doi:http://dx.doi.org/10.1007/s00112-011-2453-z
99. Network NNR, National Heart L, Institute B, Resources NCfR, Health EKSNIoC, Development H (2005) Surfactant Positive Airway Pressure and Pulse Oximetry Trial. https://ClinicalTrials.gov/show/NCT00233324,
100. Network NNR, Resources NCfR (2000) Early Surfactant to Reduce Use of Mechanical Breathing in Low Birth Weight Infants. https://ClinicalTrials.gov/show/NCT00005774,
101. Network VO (2003) Delivery Room Management Trial of Premature Infants at High Risk of Respiratory Distress Syndrome. https://ClinicalTrials.gov/show/NCT00244101,
102. Rojas MA, Lozano JM, Rojas MX, Rondon MA, Charry L, Laughon M, Bose C, Bastidas J, Ovalle O, Perez LA, Rojas C, Garcia J, Celis A, Jaramillo ML (2007) Very early surfactant without mandatory ventilation

in premature infants treated with early continuous positive airway pressure. A randomized controlled trial. Acta Paediatr 96:235-235
103. S.p.A. CF (2007) Efficacy of Combining Prophylactic Curosurf With Early Nasal CPAP in Delivery Room: the Curpap Study. https://ClinicalTrials.gov/show/NCT00501982,
104. Sandri F, Ancora G, Lanzoni A, Tagliabue P, Colnaghi M, Ventura ML, Rinaldi M, Mondello I, Gancia P, Salvioli GP, Orzalesi M, Mosca F, Italian Soc N (2004) Prophylactic nasal continuous positive airways pressure in newborns of 28-31 weeks gestation: multicentre randomised controlled clinical trial. Archives of Disease in Childhood 89 (5):F394-F398. doi:10.1136/adc.2003.037010
105. Sandri F, Plavka R, Simeoni U (2008) The CURPAP study: An international randomized controlled trial to evaluate the efficacy of combining prophylactic surfactant and early nasal continuous positive airway pressure in very preterm infants. Neonatology 94 (1):60-62. doi:http://dx.doi.org/10.1159/000113060
106. Simeoni U, Sandri F, Plavka R (2009) CURPAP study - An international randomised controlled trial to evaluate the efficacy of combining prophylactic surfactant and early nasal continuous positive airway pressure in extremely low gestational age neonates. Neonatology 95 (4):378. doi:http://dx.doi.org/10.1159/000209304
107. Imani M, Derafshi R, Arbabisarjou A (2013) Comparison of nasal continuous positive airway pressure therapy with and without prophylactic surfactant in preterm neonates. Intensive Care Medicine 39:S138. doi:http://dx.doi.org/10.1007/s00134-013-2950-8
108. Merritt TA, Hallman M, Bloom BT, Berry C, Benirschke K, Sahn D, Key T, Edwards D, Jarvenpaa AL, Pohjavuori M, et al. (1986) Prophylactic treatment of very premature infants with human surfactant. N Engl J Med 315 (13):785-790. doi:10.1056/nejm198609253151301
109. Thomson MA, Grp IS (2002) Early nasal continuous positive airways pressure (nCPAP) with prophylactic surfactant for neonates at risk of RDS. The IFDAS multi-centre randomised trial. Pediatric Research 51 (4):379A-379A
